# New insights for titanium(iv) speciation in acidic media based on UV-visible and ^31^P NMR spectroscopies and molecular modeling†

**DOI:** 10.1039/d1ra04284j

**Published:** 2021-08-09

**Authors:** Lucas Mangold, Hubert Halleux, Sébastien Leclerc, Aurélien Moncomble, Gérard Cote, Alexandre Chagnes

**Affiliations:** Université de Lorraine, CNRS, GeoRessources F-54000 Nancy France alexandre.chagnes@univ-lorraine.fr; Prayon Rue Joseph Wauters 144 à B-4480 Engis Belgium; Université de Lorraine, CNRS, LEMTA F-54000 Nancy France; Univ. Lille, CNRS, UMR 8516, LASIRE-Laboratoire Avancé de Spectroscopie pour Les Interactions La Réactivité et L’Environnement F-59000 Lille France; PSL Research University, Chimie ParisTech, CNRS, Institut de Recherche de Chimie-Paris (IRCP) F-75005 Paris France

## Abstract

Titanium chemistry in aqueous acidic media has been extensively investigated over the last decades. Hydrolyzed species such as Ti(OH)^3+^, TiO^2+^, Ti(OH)_2_^2+^ or Ti(OH)_3_^+^ have been identified and their equilibria have been studied in nitric and perchloric acid. A predominance of the divalent cations was found for low pH (*i.e.*, pH <2). Nonetheless, recent literature reports the existence of small titanium oxo-clusters in aqueous acidic media for large titanium(iv) concentration (typically., >0.1 mol L^−1^), as stable precursors for the formation of condensed titanium dioxide. The present paper reconsiders firstly previous knowledge about the speciation of titanium(iv) in non-complexing acidic media by giving evidence for the presence of polynuclear hydrolyzed species, even at very low Ti(iv) concentration (*i.e.*, typically <0.1 mmol L^−1^). UV-visible absorbance spectra recorded for diluted nitric acid solutions (a model of non-complexing acidic medium) containing titanium(iv) were compared to time-dependent density functional theory (TD-DFT) predicted excitation energies. Experimental and predicted maximal absorbance wavelengths showed significantly improved matches when polynuclear species were considered in TD-DFT calculation. Then, 0.1–12.7 mol L^−1^ phosphoric acid solutions containing titanium(iv) were studied by means of spectroscopic techniques (UV-visible, NMR) in order to identify qualitatively the presence of titanium(iv) complexes and to link this speciation to the acid concentration. Two different titanium(iv) orthophosphate complexes, potentially polynuclear, were detected, and the presence of free titanium(iv) is also expected for low phosphoric acid concentration (*i.e.*, <0.1 mol L^−1^). A general complexation scheme for a large range of H_3_PO_4_ concentration was thus formulated.

## Abstract 2

A spectroscopic study of titanium(iv) speciation in diluted nitric acid (model of non-complexing medium) and 0.1–12.7 mol L^−1^ phosphoric acid aqueous solutions. Evidence for the presence of polynuclear species is supported by molecular modeling.

## Graphical abstract

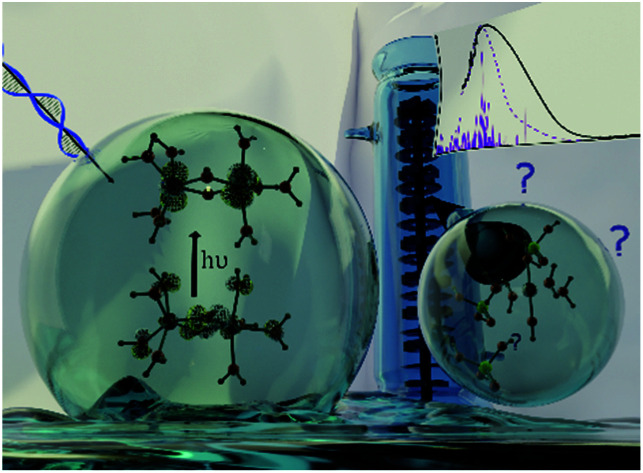

## 1. Introduction

The wet phosphoric process produces different grades of phosphoric acid (known as “wet” phosphoric acid by opposition to H_3_PO_4_ produced by pyrometallurgy) and phosphate salts that can be used in the agriculture sector (fertilizers), in food, or in the pharmaceutical or electronic industries. This process is based on the leaching of the phosphate rock with concentrated sulfuric acid and a series of purification steps to reach the purity specification requested by the target application.^1–3^ The purification steps aim at removing inorganic anions such as fluorides, sulfates, chlorides and metal impurities including cadmium, iron or titanium. For this goal, the most appropriate technology at the industrial scale is solvent extraction.^4–10^ This technology relies on the use of an extraction solvent, which can efficiently extract phosphoric acid from the leach solution. However, most common solvents exhibit a lack of selectivity against several impurities including titanium(iv).^11,12^ In order to remove titanium from wet phosphoric acid, it is therefore mandatory to implement additional purification steps.

The design of extraction solvents selective of H_3_PO_4_ against titanium is challenging. It requires knowing finely the speciation of titanium(iv) in concentrated phosphoric acid and its liquid–liquid extraction equilibria. UV-visible,^12–14^ Raman^14^ and NMR spectroscopies,^15^ liquid–liquid extraction,^16^ solubility measurements^17,18^ and potentiometric methods^19^ were used to investigate the speciation of titanium(iv) in various media such as nitric, perchloric, sulfuric, hydrochloric and phosphoric acids. The divalent titanium(iv) aquocation was generally found to be predominant in perchloric or nitric acid solutions in the absence of any complexing agents.^13,16,18^ The exact structure of this divalent cation is still debated as it was initially identified as a titanium oxocation (titanyl TiO^2+^)^20^ whereas more recent studies suggested an intramolecular rearrangement leading to the formation of titanium(iv) dihydroxide [Ti(OH)_2_]^2+.^^15,21,22^ According to the most recent literature, the hydrolysis equilibria of titanium(iv) in non-complexing acidic media can be written as follows:

1a [Ti(OH)_4_](aq.) + H_3_O^+^ ⇌ [Ti(OH)_3_]^+^(aq.) + 2H_2_O (*K*_H,1_)

1b [Ti(OH)_3_]^+^(aq.) + H_3_O^+^ ⇌ [Ti(OH)_2_]^2+^(aq.) + 2H_2_O (*K*_H,2_)

1c [Ti(OH)_2_]^2+^(aq.) + H_3_O^+^ ⇌ [Ti(OH)]^3+^(aq.) + 2H_2_O (*K*_H,3_)

The corresponding hydrolysis constants are reported in Table 1.

**Table 1:** Equilibrium constants as log *K* for Ti(iv) hydrolysis reactions at 25 °C (reproduced with permission from Elsevier from Pichavant *et al.*^21^). ND = not determined in the original papers

Hydrolysis reactions	Liberti^16^	Nabivanets^23^	Lobanov^24^	Nazarenko^25^	Ziemniak^17^	Schmidt^18^	Janaf database^26,27^
Ti^4+^(aq.) + H_2_O ⇌ Ti(OH)^3+^(aq.) + H^+^	ND	2.79	−0.16	0.097	ND	ND	3.75
Ti(OH)^3+^(aq.) + H_2_O ⇌ Ti(OH)^2+^_2_(aq.) + H^+^	−1.8	2.35	−0.59	−0.31	ND	ND	3.26
Ti(OH)^2+^_2_(aq.) +H_2_O ⇌ Ti(OH)^+^_3_(aq.) + H^+^	−2.4	−2.1	−0.92	−0.64	−2.28	−1.85	−1.71
Ti(OH)^+^_3_(aq.) + H_2_O ⇌ Ti(OH)_4_(aq.) + H^+^	−2.1	−3.37	1.05	−0.96	−1.9	−2.95	−3.07

In the presence of inorganic ligands in solution, titanium(iv) can form various complexes as described in eqn (2) in the particular case of acidic sulfate solutions:

2 [Ti(OH)_2_]^2+^(aq.) + *n*H_*m*_SO^*m*−2^_4_(aq.) ⇌ [Ti(OH)_2_(H_*m*_SO_4_)_*n*_]^2−*n*(2−*m*)^(aq.)

where *m* = 0 or 1 and *n* = 1 or 2, and the corresponding equilibrium constant is expressed as follows:3
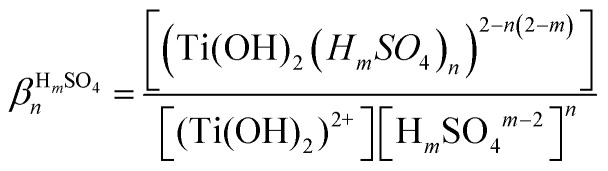


Table 2 evidences significant discrepancies between the formation constants *β*_*n*_^H_*m*_SO_4_^ in the presence of hydrogen sulfate and sulfate ions.^21^ More recently,^28^ investigation of TiOSO_4_ dissolution in water and in 1.0 mol L^−1^ HNO_3_ by X-ray absorption spectroscopy and small angle X-ray spectroscopy showed polycondensation phenomena responsible for the possible presence of [Ti_18_O_27_(SO_4_)_9.5_]^−^ anions at titanium(iv) concentrations ranging between 0.25 mol L^−1^ and 1 mol L^−1^. Likewise, other studies showed the presence of small titanium clusters whose size changed depending on the experimental conditions.^29–32^ Such polytitanates species with bridging oxo groups were also identified in anhydrous conditions in the case of titanium alkoxide compounds such as titanium ethoxide.^33,34^ Therefore, it cannot be excluded that polycondensation of titanium(iv) also occurs in phosphoric acid. However, only few studies concern the investigation of the speciation of titanium(iv) in phosphoric acid and the conclusions of these studies are still subject of controversies.^19,35,36^

**Table 2:** Equilibrium constants for complexation of Ti(iv) (log *β*_*n*_) by hydrogen sulfate and sulfate at 25 °C. Two constants are available for Szilágyi *et al.*^37^ depending on the nature of the added salt: (a) Na_2_SO_4_ (b) (NH_4_)SO_4_ (reproduced with permission from Elsevier from Pichavant *et al.*^21^). ND = not determined in the original papers

		Nabivanets^38^	Babko^39^	Baillon^40^	Szilágyi^37^	
SO_4_^2−^	log *β*1^SO_4_^	2.4	2.23	0.53	0.85 (a)	0.99 (b)
	log *β*2^SO_4_^	3.6	4.12	1.08	0.68 (a)	0.97 (b)
	log *β*3^SO_4_^	ND	4.25	ND	ND	ND
HSO_4_^−^	log *β*1^HSO_4_^	2.15	ND	0.04	ND	ND
	log *β*2^HSO_4_^	ND	ND	−0.04	ND	ND

To gain a better knowledge of titanium(iv) chemistry in acidic aqueous media, we have decided to reinvestigate as a starting point the speciation of Ti(iv) in dilute nitric acid solution (*i.e.*, [HNO_3_] <2 mmol L^−1^ HNO_3_) as a model of non-complexing acidic media. For this goal, UV-visible spectroscopy was used and completed with time-dependent density function theory calculations (TD DFT). Then, the speciation of Ti(iv) was investigated at its turn in a large range of concentration of phosphoric acid (*i.e.*, in 0.1–12.7 mol L^−1^) by ^31^P NMR spectroscopy and UV-visible spectroscopy coupled with chemometric analyses.

## 2. Experimental section and DFT calculation

### 2.1 Materials and methods

#### Preparation of orthotitanic acid (H_4_TiO_4_)

All work was carried under a fume hood. Orthotitanic acid (H_4_TiO_4_) used to prepare a mother solution of various Ti(iv) was obtained as follows. A volume of 5 mL of titanium tetraisopropoxide (Sigma Aldrich, purity = 97%) was added dropwise to 25 mL of ethanol (Carlo Erba, 96%) at 0 °C under constant stirring. A white precipitate of orthotitanic acid was formed immediately.^41^ Afterwards, a mixture of 25 mL ethanol (Carlo Erba, 96%) and 3 mL of deionized water cooled at 0 °C (resistance = 18.2 Ω) was then added to the orthotitanic acid suspension. After 1 h of agitation in an ice bath, the suspension was filtered under vacuum by means of a fritted glass filter (cat. 4). The solid was dried for 1 h under vacuum at room temperature (the solid was regularly triturated during drying). A white powder (∼3 g) of titanium hydroxide was obtained.

#### Dissolution of titanium(iv) in nitric acid

All work was carried under a fume hood. Approximately 0.3 g of the orthotitanic acid was dispersed in 10 mL of 0.73 mol L^−1^ nitric acid prepared by dilution of 65% (vol) nitric acid (analytical grade, Merck) in deionized water. After 24 h under agitation, orthotitanic acid was almost completely dissolved in nitric acid. In order to complete the dissolution, 20 mL of 0.73 mol L^−1^ nitric acid was added again. Residual insoluble powder of orthotitanic acid was removed by filtration under vacuum with a fritted glass filter (cat. 4). The turbid solution was then filtered using an Acrodisc Supor syringe filter containing a PES membrane (porosity = 0.2 μm, Pall) previously conditioned by using 0.73 mol L^−1^ nitric acid. The titanium concentration in the filtrate was determined by using an Agilent Microwave-Plasma Atomic Emission Spectrometer (see below). Titanium concentration was found to be equal to 2.3 g L^−1^.

#### Dissolution of titanium(iv) in phosphoric acid

All work was carried under a fume hood. Approximately 2.7 g of orthotitanic acid was added under agitation to 30 mL of 85% (weight) phosphoric acid (Sigma-Aldrich). After one night of agitation, a small amount of deionized water was added to decrease the viscosity. The suspension was filtered under vacuum with fritted glass filter (Cat. 4) previously conditioned with deionized water. Titanium concentration in the filtrate was determined by using the Agilent Microwave-Plasma Atomic Emission Spectrometer (the filtrate was still slightly turbid). The titanium(iv) concentration was found to be equal to 8.0 g L^−1^.

#### Potentiometric titrations

The nitric acid concentration in the diluted nitric acid stock solution was determined by potentiometry with 0.1 mol L^−1^ sodium hydroxide (VWR, AVS Titrinorm, ±0.2%). The pH-meter used for the potentiometric titration was a pHenomenal pH 1100 L (VWR) equipped with a pHenomenal pH electrode. The pH electrode was calibrated by using two buffer solutions at pH = 4 and pH = 7 (VWR, AVS Titrinorm). The concentrations of phosphoric acid in the stock solution and in the titanium solutions were also determined by potentiometry with a fresh solution of 0.1 mol L^−1^ sodium hydroxide (Merck, EMPROVE Expert ±2.5%). The same pH-meter and the same electrode were used but three buffer solutions at pH = 4, pH = 7 and pH = 9 (VWR, AVS Titrinorm), respectively, were used to calibrate the pH electrode.

#### Elemental analyses

Titanium concentrations in the samples were determined by using an Agilent Microwave-Plasma Atomic Emission Spectrometer (wavelength = 323, 452 nm). The mother standard solution containing 1000 ppm of titanium in 2% (vol) nitric acid (Sigma Aldrich, ICP Standard) was diluted in 5% (vol) nitric acid to prepare five standards containing 1 to 10 ppm titanium. In order to minimize possible matrix effects, two different series of standards were prepared, without or with H_3_PO_4_.

#### UV-visible spectroscopy

An Agilent Cary 60 UV-visible spectrophotometer was used to record spectra between 190 nm and 400 nm with a step of 0.5 nm. A 2 mm light path cuvette (Agilent) was used to analyze the solutions containing less than 1 mmol L^−1^ titanium. A 0.1 mm light path demountable cuvette (Hellma Analytics) was used to analyze the solutions containing titanium at concentration greater than 1 mmol L^−1^.

In nitrate media, a good signal-to-noise ratio was obtained providing that the nitrate concentration was lower than 2 mmol L^−1^ as the nitrate ion exhibits a broad absorption peak at 200 nm with a molar absorption coefficient approximately equal to 10^5^ cm^−1^ L mol^−1^. Therefore, the titanium(iv) solutions were diluted and the [Ti]/[NO_3_] ratio was controlled by adding the requested amount of nitric acid in the solutions. The reference solutions were prepared by using the same protocol. The blank was 0.73 mol L^−1^ nitric acid, which was filtered by using a 0.2 μm syringe filter with PES membrane prior to UV-vis analyses.

#### ^31^P NMR spectroscopy

^31^P NMR spectra were recorded at 161.975 MHz on a Bruker AVANCE III 400 spectrometer at 25 °C equipped with a 5 mm BBFO broad-band probe. Samples were introduced in a 5 mm NMR tube with a coaxial insert containing D_2_O as a lock solvent. The D_2_O is separated from the samples in order to avoid affecting the pH of the solution. Chemical shifts are referred to external H_3_PO_4_ (0 ppm). ^31^P NMR data were acquired with 128 scans, 64 *k* points, a repetition delay of 2 s and a spectral width of 16 164 Hz.

### 2.2 Computational methods

#### Chemometric analysis

The R2019b version of Matlab was used for chemometric analyses of the spectral dataset. This analysis relies on the determination of the Malinowski factor indicator function criterion.^42–44^ It was used to determine the number of chromophores detectable within a given range of concentrations and wavelengths as reported by Meloun *et al.*^45^ This criterion relies on a component analysis calculated from the covariance matrix 𝕊𝕊^T^. The eigenvalue vector of this covariance matrix gives information about the components associated to independent signals in the UV-visible spectra. The Malinowski factor indicator function IND criterion is calculated by using eqn (4) for different *k*-values, which correspond to the number of chromophores in solution. The number of chromophores is equal to the value of *k* corresponding to the minimum value of IND.4
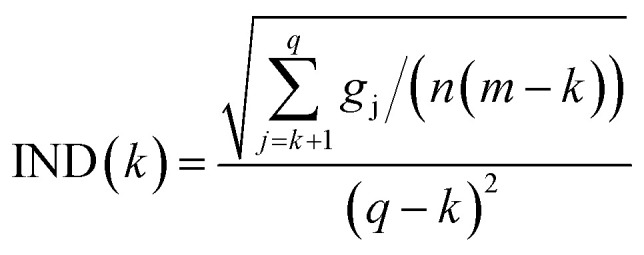
where *g*_j_, *n* and *m* denote the *j*-th eigenvalue of the covariance matrix, the number of spectra used in the dataset and the number of wavelengths, respectively. The *q* parameter is equal to the highest value between *n* and *m*.

To better identify the number of chromophores in solution from the spectral data, a common data analysis procedure was used. Numerical noise (jitter) was added to the recorded spectra in order to erase the contribution of the minor components and to distribute more uniformly noise in the considered wavelength range. Although jittering is usually used to reduce overplotting and better identify clusters in large datasets,^46^ it was found efficient to identify the number of chromophores.

#### DFT calculations

Time independent and time dependent density functional theory (DFT and TD-DFT) calculations were performed using the Gaussian package (version 16).^47^ The structures considered in this work were optimized geometrically with the PBE0 global hybrid functional^48,49^ and the 6-311+G(d,p) basis set.^50–52^ The solvation of the first coordination sphere was described explicitly whereas the outer solvation sphere was described by using the PCM model.^53,54^ Frequencies were calculated on the optimized geometries. Excitation energies were then determined by TD-DFT with the same level of theory with a linear response non equilibrated PCM solvation model with water. Unless otherwise stated, a total number of 80 states were computed in order to get a representative range of the experimental wavelength where light absorption occurred. The same type of calculation was performed on a larger system, *i.e.* a titanium oxo-cluster denoted Ti_18_, and the procedure and parameters (basis set, number of computed states and solvation model) used for this system is detailed in the ESI.†

The use of hybrid functionals as well as the implicit solvation model PCM was found to be efficient to obtain reliable estimations of the wavelengths of maximal absorbance for metal complexes,^55^ molecular systems in complex environments^56^ as well as polyoxometalates.^57^ This scheme and the pitfalls of the implemented methods have been well-known for a decade.^58^ In the current state of the art, the computed excitation energies may thus be considered reasonably reliable as illustrated by our previous works using the same methodology.^59–61^

The orbital transitions associated with the main predicted excitation energies were described by calculating natural transition orbitals (NTO).^62^

The structures of the optimized geometries and the representation of the Natural Transition Orbitals (NTOs) were generated using Avogadro^63^ software and POV-Ray^64^ as post-treatment (ray-tracer).

## 3. Results and discussion

### 3.1 Titanium(iv) speciation in dilute nitric acid (non-complexing media)

#### 3.1.1. UV-visible spectroscopy

**Fig. 1:**
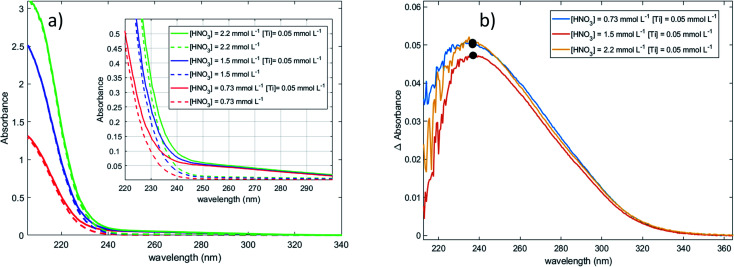
UV-vis Spectra (after baseline correction) of (a) 0.05 mmol L^−1^ of titanium(iv) in 0.73–2.2 mmol L^−1^ nitric acid (solid lines) and aqueous solutions of 0.73–2.2 mmol L^−1^ nitric acid (dotted lines) recorded with a 2 mm light path cuvette, and (b) difference between the spectra of Ti(iv)–HNO_3_ and the spectra of HNO_3_.

UV-visible spectra of 0.05 mmol L^−1^ titanium(iv) in 0.73–2.2 mmol L^−1^ nitric acid were recorded in order to investigate the speciation of titanium(iv) [Fig. 1a]. It is expected that no complexation of titanium(iv) occurs in such nitric acid solutions as nitrate ions exhibit only a weak tendency to form nitrato complexes with most metal ions.^65–68^ For instance, the formation constants of mono manganese and cadmium nitrate complexes (MNO_3_^+^ with M = Mn or Cd) at 298.6 K are equal to 0.17 and 0.54, respectively.^67^ Likewise, the formation constants of niobium(iii), thorium(iv) and uranium(vi) nitrate complexes at 298 K are equal to 0.64 at ionic strength *I* = 2 mol L^−1^, 0.22 (*I* = 1 mol L^−1^) and 0.24 (*I* = 1 mol L^−1^), respectively.^65,66,68^ In the present work, it can be expected that only free titanium(iv) exists in solution^69^ as nitrate concentration is low, *i.e.* [HNO_3_] = 0.73–2.2 mmol L^−1^.

Fig. 1a shows the presence of an intense absorption in the UV region.^13,14,70^ The strong band located between 180 and 220 nm (region 1) can be attributed to nitrate ions,^71^ and the band which appears in the range 220–350 nm (region 2) on Fig. 1b after baseline correction may correspond to the presence of one or several titanium(iv) complexe(s). In coherence with the above statement, it is interesting that the intensity of the band located in region 1 strongly and almost linearly increases with the concentration of nitric acid whereas it does not depend on the presence or absence of titanium(iv). On the opposite, the intensity of the band located in region 2 (Fig. 1b) is independent on the concentration of nitric acid within the experimental uncertainty, which confirms the absence of nitrato complexes of Ti(iv) in 0.73–2.2 mmol L^−1^ HNO_3_ in agreement with Szilágyi *et al.*^44^. The noise observed in Fig. 1b for the shortest wavelengths is due to the strong absorbance of nitrate ions around 200 nm.

The large width of the absorbance peak for wavelengths between 230 and 275 nm suggests an asymmetry with respects to the region between 200 and 230 nm. This may result from the presence of several titanium(iv) complexes in solution or the presence of a unique titanium(iv) complex exhibiting several major possible electronic transitions.

**Fig. 2:**
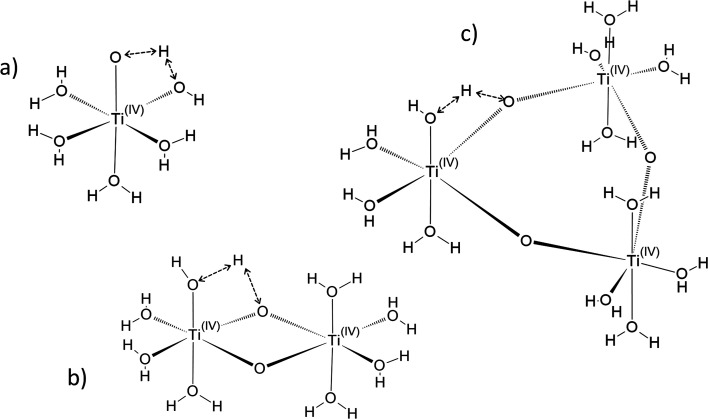
Schematic structures suggested for the free form of titanium [(TiO)_*μ*_]^2*μ*^ considering various sizes, *i.e.* monomeric (a, *μ* = 1), dimeric (b, *μ* = 2), trimeric (c, *μ* = 3). Possible intramolecular proton exchanges are represented by dashed double arrows.

In the following part of the paper, titanium(iv) species are written as [(TiO)_*μ*_]^2*μ*^ with *μ* = 1 for mononuclear species and *μ* >1 for polytitanate species as it is not possible to exclude the presence of polynuclear species in solution. For *μ* >1, as illustrated in Fig. 2, an octahedric structure with bonding oxo groups is suggested. A similar proton exchange equilibrium as TiO^2+^ ⇌ Ti(OH)_2_^2+^ for mononuclear species might occur in the case of polynuclear species, leading to the formation of hydroxo bridging groups (see Fig. 2).

#### 3.1.2. DFT calculations

To get information about the chemical structure and the stoichiometry of the titanium(iv) species in non-complexing media, computed transition energies of several complexes were obtained by Time-Dependent Functional Density Theory (TDDFT) calculations.

**Fig. 3:**
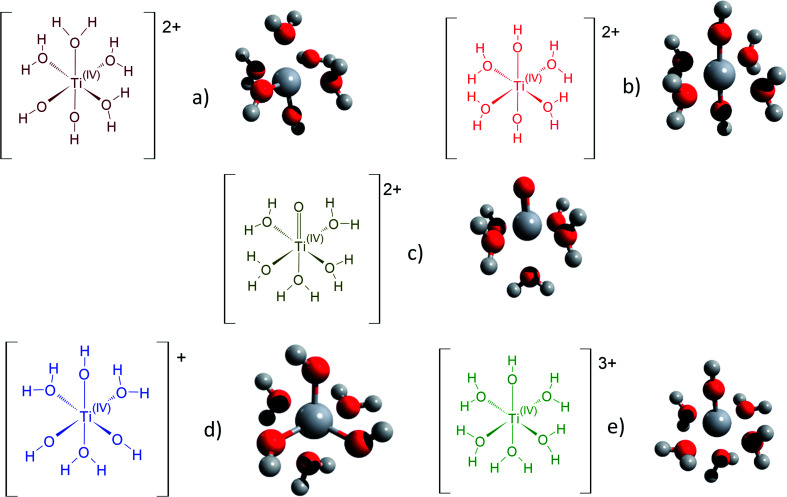
Schematic representation of the first hydratation shell and optimized geometries for (a) [Ti(OH)_2_]^2+^(cis) (b) [Ti(OH)_2_]^2+^ (trans) (c) [TiO]^2+^ (d) [Ti(OH)_3_]^+^ (e) [Ti(OH)]^3+^. Cartesian coordinates are available as part of the ESI.†

Fig. 3 gathers the different configurations reported in the literature of free titanium in acidic medium [(TiO)_*μ*_]^2*μ*^ (geometry optimized by DFT calculations in the present work).^13,16,18,22^ Both TiO^2+^ and Ti(OH)_2_^2+^ cations as well as Ti(OH)_3_^+^ and Ti(OH)^3+^ were considered in order to figure out the predominant species in solution. The starting geometries were thus obtained by placing one to three hydroxyl groups (and oxo group for TiO^2+^) coordinated to titanium(iv) cation. The rest of the octohedra sites of the complexes were filled with water molecules (solvation shell). The outer solvation shells were described by an implicit model of water molecules (PCM) in accordance with other DFT works, which demonstrated that such kind of models are relevant to describe transition metal complexes in aqueous media.^61,72^

The structures used in the TD-DFT calculations were local potential energy minima, as the vibration modes were all associated with positive eigenvalues after geometry optimization. The octahedricity was conserved during the optimization even though an important distortion was observed for the Ti(OH)_3_^+^ and Ti(OH)_2_^2+^ cis structures as shown in Table 3. The resulting Ti–(OH_2_), Ti=(O) and Ti–(OH) bond distances of the final geometrically optimized structures reported in Fig. 3 are gathered in Table 3.

**Table 3:** Typical bond lengths (in angstroms) and angles (in degrees) of optimized geometries for structures in Fig. 3

	TiO^2+^	Ti(OH)_2_^2+^ (cis)	Ti(OH)_2_^2+^ (trans)	Ti(OH)^3+^	Ti(OH)_3_^+^
Mean Ti–H_2_O distance	2.07	2.11	2.05	2.02	2.22
Axial Ti–H_2_O distance (TiO^2+^ and Ti(OH)^3+^)	2.29	n.a.	n.a.	2.09	n.a.
Ti=O, Ti–OH distance (TiO^2+^ and Ti(OH)^3+^)	1.58	n.a.	n.a.	1.68	n.a.
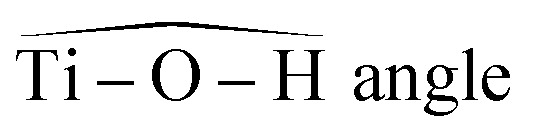	n.a.	146°	173°	180°	128°
Mean Ti–OH distance	n.a.	1.74	1.76	n.a.	1.79
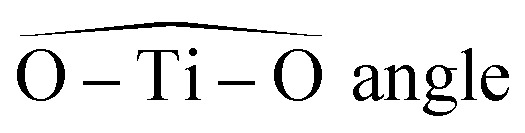 (minimal and maximal values)	82°, 97°	75°, 105°	89°, 91°	80°, 100°	75°, 105°

The excitation energies of the optimized complexes were calculated using TD-DFT (excited states are available in the ESI†). To do so, the scheme described in the Computational Section was used to predict UV-visible spectra as its accuracy generally allows to compute transitions within 0.25 eV, which is sufficient in the case of the qualitative approach of this work.

**Fig. 4:**
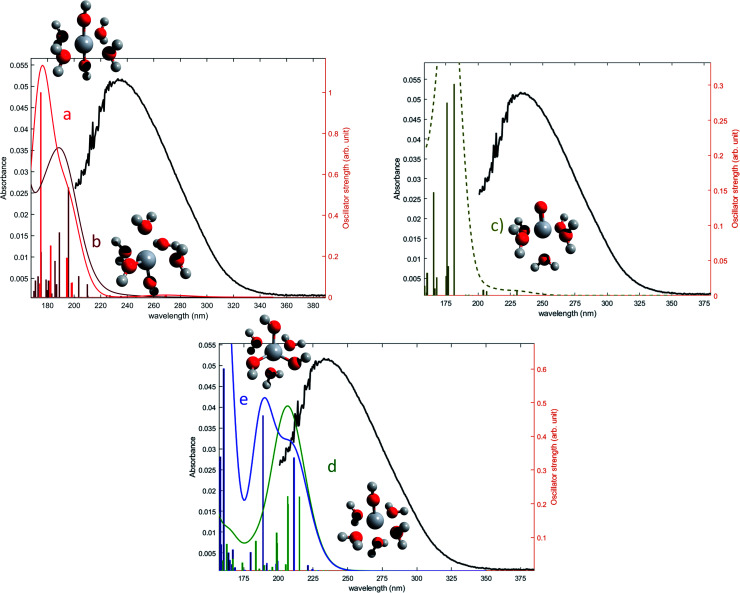
Comparison between predicted excitation energies with their relative associated oscillator strength (absolute values available in ESI†) for (a) [Ti(OH)_2_]^2+^(cis) (b) [Ti(OH)_2_]^2+^ (trans) (c) [TiO]^2+^ (d) [Ti(OH)_3_]^+^ (e) [Ti(OH)]^3^ and the spectrum of free titanium (1b).

The oscillator strengths associated with the permitted transitions are shown in Fig. 4. This value represents the probability of a transition, and thus it might be compared to an absorbance measurement for the wavelength corresponding to the transition energy. UV-visible plots were obtained by convolving the transitions with a Gaussian function (assuming a standard deviation of 0.4 eV) to account for the band shape. The excitation energies and associated oscillator strengths, as well as the reconstructed spectra were compared to the experimental spectrum of free titanium reported in the first part of the discussion. For each mononuclear complex considered in these calculations, no agreement was observed between the most relevant transitions and the experimental location of the maximum of absorbance (235 nm). The computed transitions are significantly blue-shifted with respect to the experimental data whatever the structure considered. Therefore, the presence of any of these divalent mononuclear titanium(iv) [Ti(OH)_2_]^2+^ in acidic nitrate solutions cannot be supported by this study. In other words, the free titanium(iv) complex in non-complexing acidic solutions (perchloric or nitric acid) may not match with [Ti(OH)_2_]^2+^ cation as it was previously stated in the literature.^13,15,16,18^

**Fig. 5:**
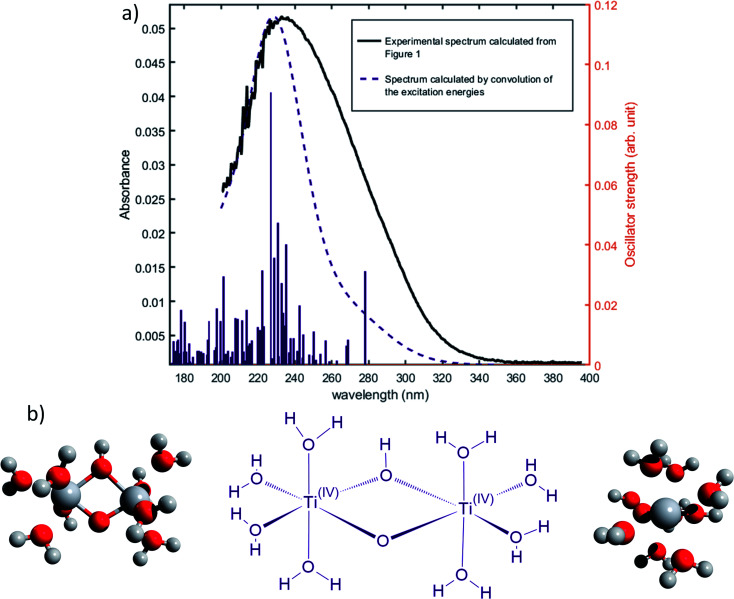
(a) Predicted transitions and their associated oscillator strengths for a dimeric structure [Ti_2_(μ-(OOH))]^5+^ compared to the experimental spectrum of free titanium calculated in 1b. The convolution of excitation energies by gaussians (wavenumber standard deviation of 0.4 eV) is plotted with a dotted line. (b) Optimized geometry (upper and lateral view) and schematic representation of a dimeric polytitanate [Ti_2_(μ-(OOH))]^5+^.

The formation of polytitanate species is suspected even in acidic solutions. The computational cost of the level of theory chosen to perform the calculation (6-311+G**/PBE0) was a limiting factor to perform the geometry optimization on large oxoclusters. Thus, the excitation energies were obtained only for the dimeric form of titanium aquo-complexes. Different structures containing several hydroxyl groups coordinated to titanium(iv) atoms were considered. Two bridging oxygen atoms were inserted between the titanium atoms and their three different protonation states were chosen for calculations. The spectra of the dimeric structures showed a fair agreement with the experimental spectrum obtained in diluted nitric acid (Fig. 5a). The best agreement between the experimental and calculated UV-visible spectra was obtained for the chemical structure displayed in Fig. 5b. The 3D representation shows that this optimized structure consists of two titanium(iv) atoms bonded *via* a mixed μ-(OOH) bridge. The first coordination spheres of the two titanium(iv) cations were completed with water molecule as for the monomeric complexes. However, the shape of the band between 230 nm and 340 nm is poorly reproduced. It is worth mentioning that a predicted transition associated with a non-negligible oscillator strength is present at 275 nm as depicted in Fig. 5a. The width of the experimental peak between 250 nm and 300 nm might be explained by the presence of electronic transitions at similar wavelength associated with a higher oscillator strength.

**Fig. 6:**
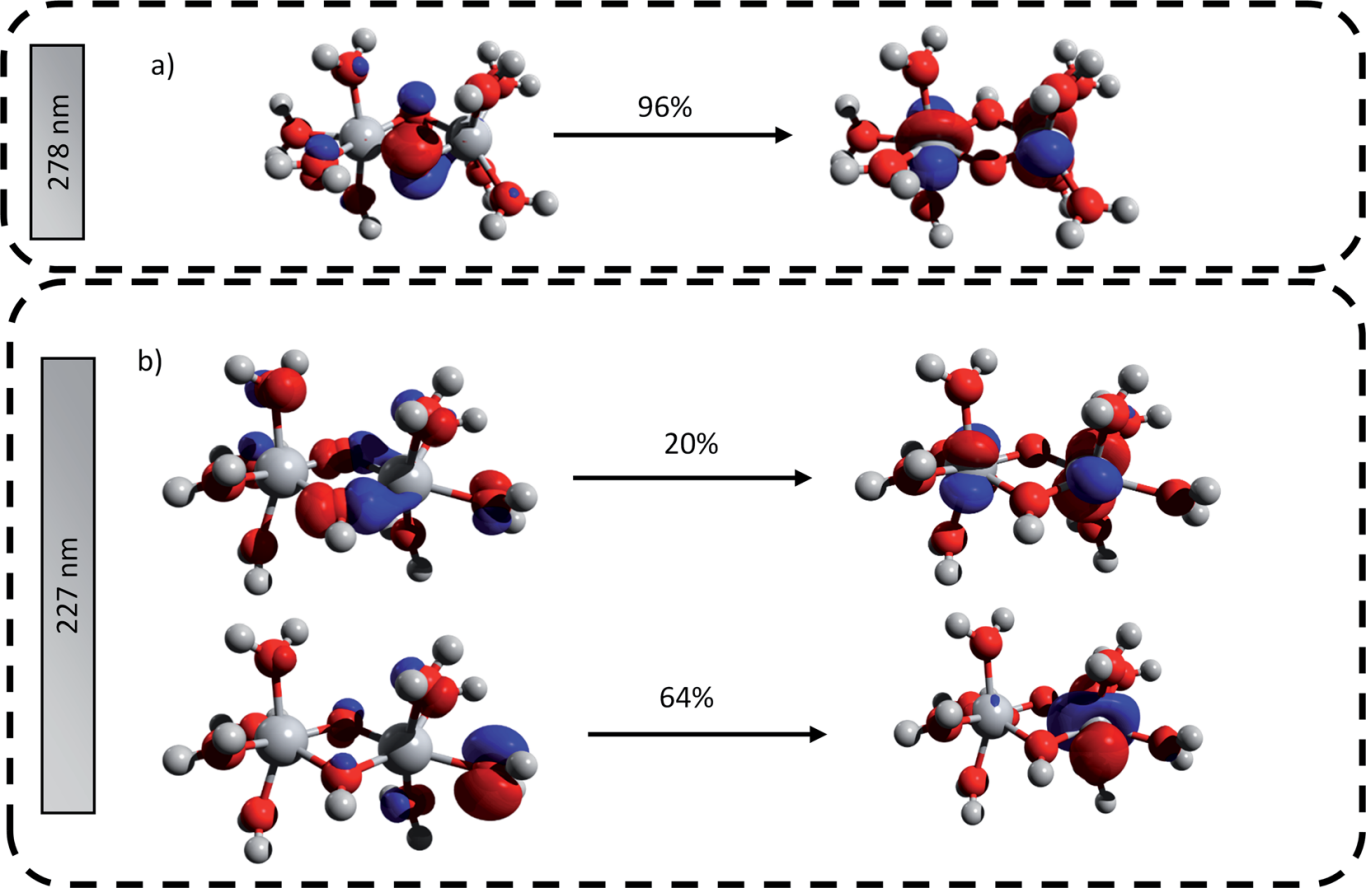
Natural transition orbitals generated for the predicted transitions at (a) 278 nm and (b) 227 nm. The digits represent the contribution in percent of the drawn NTO transitions.

The main transitions located at 227 nm and 278 nm (the most intense in their respective wavelength domains) were described with the natural transition orbitals (NTO) method (Fig. 6). The identification of the orbitals involved in the main transitions was found to be much easier with the use of the NTOs than with the standard canonical orbitals (see ESI†). The first transition (227 nm, 5.46 eV) was decomposed into two contributions: (i) an electron transfer from the p-orbital corresponding to non-bonding electrons of oxygen atoms from several coordinated water molecules to the d_*z*^2^_-orbital of one of titanium atom, and (ii) an electron transfer from p-orbitals of the two bridging oxygens and of the oxygen atoms of coordinated water molecules to the d-orbitals of titanium atoms. The second excitation (278, 4.48 eV) was described by a similar transition, *i.e.* p-orbitals of bridging oxygen atoms to d-orbitals of titanium atom. Both these descriptions support the ligand-to-metal charge transfer character of the most intense transitions in the titanium(iv) complex.

**Fig. 7:**
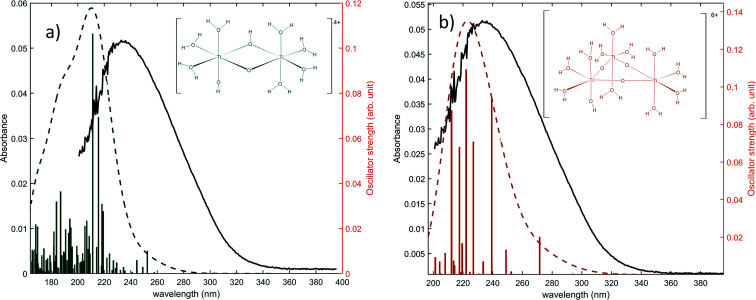
Predicted transitions and their associated oscillator strength for (a) a dimeric structure [Ti_2_(μ-(OOH)) (OH)]^4+^ (b) a trimeric structure [Ti_3_(μ-(O)_3_)]^6+^ compared with the experimental spectrum of free titanium from Fig. 1b. The convolution of excitation energies by gaussians (wavenumber standard deviation of 0.4 eV) is plotted with a dotted line.

The same type of calculation has been performed for two other titanium(iv) complexes whose chemical structure remains close to the previous one: the first one in which a water molecule in the solvation shell was replaced by an hydroxyl group (Fig. 7a), and the second one corresponding to a trimer structure with bridging oxo groups (Fig. 7b). The main excitation energy predicted for the trimer structure shows also a good agreement with the experimental wavelength. As for the second dimer structure, a difference of about 15 nm was found. In comparison to the results obtained for monomeric structures, this difference between the experimental and the calculated wavelengths remains acceptable. Therefore, TD-DFT cannot be used to conclude about the exact structure of titanium(iv) in nitric acid because of the existence of many structures whose calculated wavelengths match with experimental wavelengths.

**Fig. 8:**
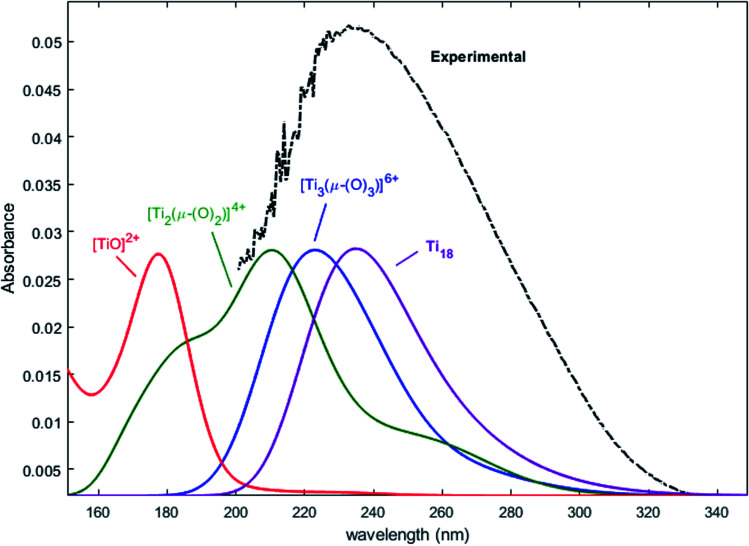
Comparison of the normalized convolution of the excitation energies calculated by TD-DFT for TiO^2+^, [Ti_2_(μ-(O)_2_)]^4+^, [Ti_3_(μ-(O)_3_)]^6+^ and Ti_18_ with the experimental UV-vis spectrum of titanium(iv) in nitric acid from Fig. 1b.

Nevertheless, this study shows that the predicted position of maximal absorbance shifts toward higher wavelengths when the size of the polyoxometalate complex species increases. This tendency was confirmed for higher nuclearity as depicted in Fig. 8, which shows the normalized convolution of the excitation energies by gaussians for TiO^2+^ as well as for oxo-bridged polytitanate species with 2, 3 and 18 titanium(iv) atoms. The cartesian coordinates of the optimized geometries of [Ti_2_(μ-(O)_2_)]^4+^ and [Ti_3_(μ-(O)_3_)]^6+^ as well as the procedure used to obtain the excited states for Ti_18_ are given in the ESI.†

The comparison with the experimental spectrum of titanium(iv) in nitric acid reported in Fig. 1b shows that the maximum of absorbance at approximately 230 nm may be linked to the presence of a “small” polytitanate species (dimeric or trimeric complex species). This Figure also shows that the width of the absorbance band in the 230–320 nm region might be due to the presence of other complex species of higher nuclearity such as the oxo-clusters suggested by Kozma *et al.*^28^

However, the comparison between the results obtained for the [Ti_2_(μ-(OOH)) (OH)]^4+^ and [Ti_2_(μ-(OOH))]^5+^ (Fig. 5 and 7) and those obtained for the [Ti_2_(μ-(OOH))]^5+^ and [Ti_2_(μ-(O)_2_)]^4+^ (Fig. 5 and 8) suggests that the presence of hydroxyl groups or water molecules coordinated to titanium(iv) and the protonation state of bridging oxo-groups may influence the position of the absorbance band.

### 3.2 Titanium(iv) speciation in phosphoric acid solutions

When phosphoric acid is added to non-complexing solutions of titanium(iv), it is expected that the [(TiO)_*μ*_]^2*μ*^ species identified above (with *μ* = 1 for mononuclear species and *μ* >1 for polytitanate species) will be converted into titanium-phosphate complexes. The goal of this part is to investigate the formation of such complexes by using ^31^P NMR spectroscopy and chemometric analyses of UV-visible spectra.

#### 3.2.1. ^31^P NMR

**Fig. 9:**
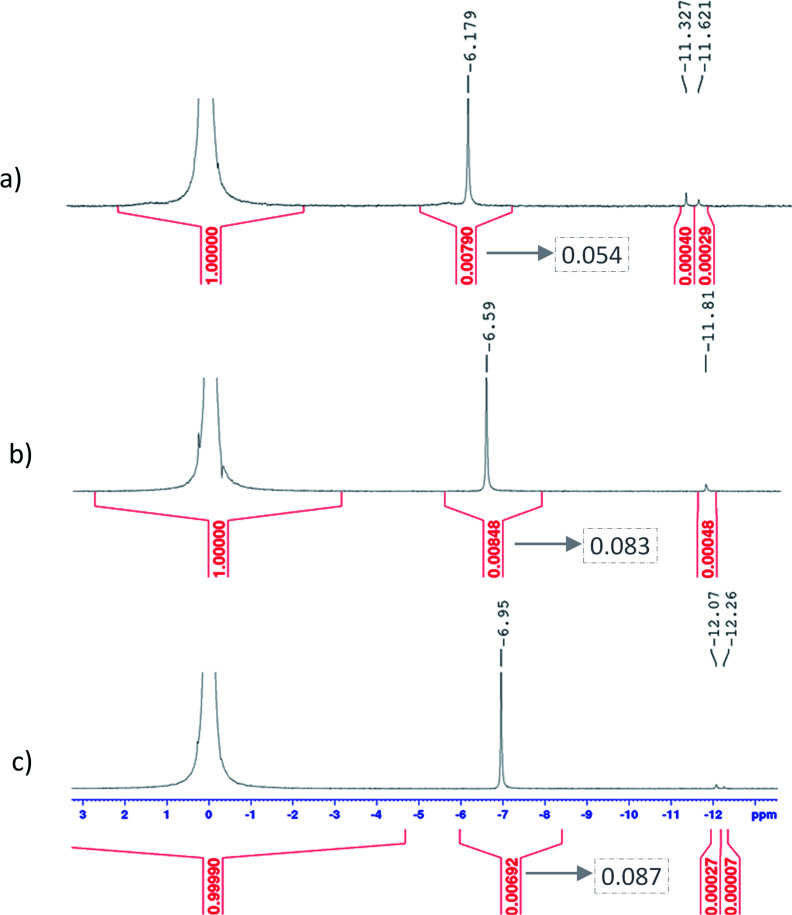
^31^P NMR spectra obtained for solutions with 17 mmol L^−1^ of titanium and a phosphoric acid concentration of (a) 6.9 mol L^−1^ (b) 9.8 mol L^−1^ (c) 12.7 mol L^−1^. The chemical shifts and integration of principal peaks are shown by black digits on top and red digits on the bottom of the spectra respectively. The digits in dotted boxes are integration values ponderated by the concentration of phosphoric acid.

Fig. 9 shows ^31^P NMR spectra of 6.9–12.7 mol L^−1^ phosphoric acid solutions containing 17 mmol L^−1^ titanium(iv). The peaks located between *δ* = −6.1 and −6.9 ppm may be attributed to a titanium–phosphate complex as the corresponding integration (after weighting) depends on the phosphoric acid concentration. In the region *δ* = −11 to −12.5 ppm, it is difficult to conclude if the two observed weak peaks correspond to (i) two different phosphorus complexes or (ii) a unique titanium–phosphate complex containing two non-equivalent phosphorus atoms characterized by two chemical shifts. Furthermore, the mean dynamic conformation could also depend on experimental conditions such as pH and viscosity correlated with phosphoric acid concentration. Previous works on the synthesis of amorphous titanium(iv) phosphate reported two peaks at similar chemical shifts obtained from the deconvolution of MAS-NMR spectra of synthesized materials (*δ* = −5.0 to −5.2 ppm and *δ* = −13.5 to −15.5 ppm).^73^ These peaks at *δ* = −5.0 to −5.2 ppm and *δ* = −13.5 to −15.5 ppm were attributed to the following types of phosphorous sites: P(OTi)_*x*_(OH)_4−*x*_ where *x* = 1 or 2 (H_3_PO_4_ or H_2_PO_4_^−^ environment) and P(OTi)_3_OH (HPO_4_^2−^ environment), respectively. Similar assignation, *i.e.* −4.5 to −7.3 ppm attributed to H_2_PO_4_^−^ and −12.5 to −15.9 ppm attributed to HPO_4_^−^ were made by Trublet *et al.*^74^ and Zhang *et al.*^75^ for amorphous titanium(iv) phosphates. The MAS-NMR spectra of a synthesized oxyfluorinated titanium(iv) phosphate^76^ led also to the conclusion that the peaks located at −7.0 ppm and −11.5 could be attributed to orthophosphates groups linked to 1 or 2 titanium(iv) centers, respectively. From these assignments, it can be assumed that the peak observed in this study between −6.18 and −6.95 ppm may correspond to a complex in which the orthophosphate group (in the form of H_3_PO_4_ or H_2_PO_4_^−^) is linked to one titanium(iv) atom. For the second region, *i.e.* −11.3 to −12.3 ppm, the peaks might be attributed to deprotonated orthophosphate groups (HPO_4_^2−^ form) in a titanium(iv) phosphate complex, possibly as bridging groups between two titanium(iv) centers.

#### 3.2.2. UV-visible spectroscopy and chemometric analysis

**Fig. 10:**
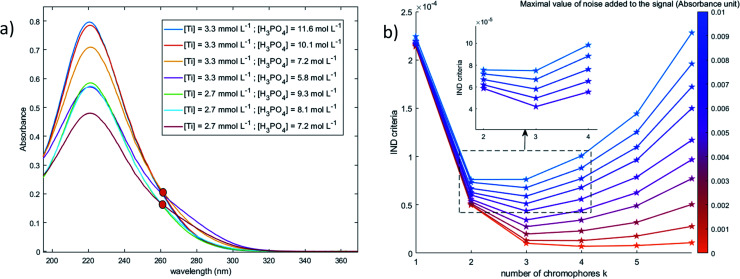
(a) Spectral dataset (after baseline correction) used for the determination of the number of chromophores (0.1 mm light path cuvette) (b) Malinowski factor indicator function calculated for different dummy value of chromophores *k* and different numerical noise addition.

Fig. 10a shows the spectral dataset of 2.6–3.3 mmol L^−1^ titanium(iv) in 5.8–11.6 mol L^−1^ phosphoric acid (after baseline correction). These spectra were used to determine the number of chromophores detectable between 200 and 400 nm by using the Malinowski factor indicator function IND criterion.^42–45^ The calculation of the IND criterion allows to identify the number of independent chromophores *k*. The orange curve (bottom) in Fig. 10b shows the variation of this criterion as a function of the hypothetical number of chromophores *k* without any external perturbation. This curve does not permit to determine the number of chromophores as it does not exhibit any minimum. Conversely, this curve reaches a plateau that can be explained by the presence of non-uniformly distributed noise as well as by the presence of species characterized by UV-visible signals of the same order of magnitude as the noise. After perturbing the signal by adding more noise, the curves exhibit a minimum value for *k* = 3, which corresponds to the number of chromophores in solutions.

The value of the Malinowski indicator function IND for the third chromophore without addition of noise is low in comparison to the value for the first and second species. Thus, even though this third species has to be taken into account to study the speciation of titanium(iv) in phosphoric acid, its contribution in the experimental spectra of Fig. 10 is expected to be negligible. Therefore, this third species will be neglected in the next part of the present paper.

**Fig. 11:**
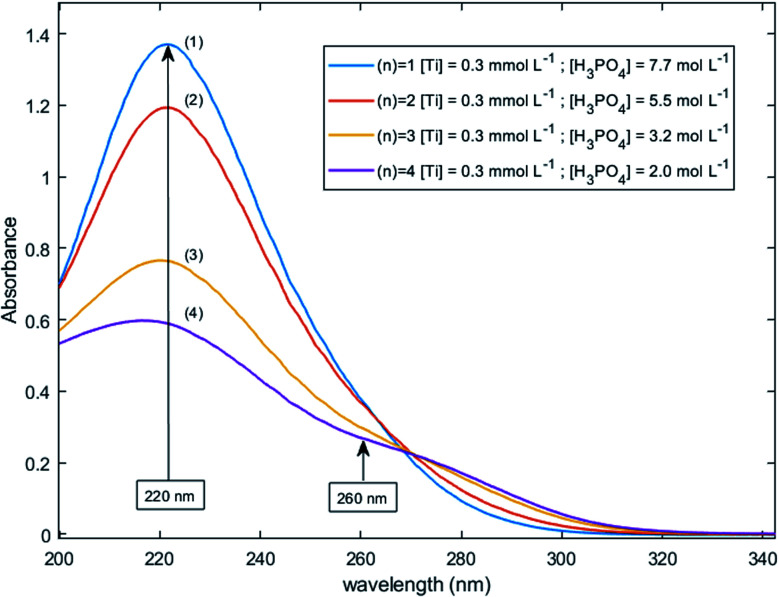
UV-vis spectra of 0.3 mmol L^−1^ Ti(iv) in 2.0–7.7 mol L^−1^ phosphoric acid after baseline correction (2 mm light path cuvette).

Fig. 11 shows UV-visible spectra of 0.3 mmol L^−1^ titanium(iv) in 2.0–7.7 mol L^−1^ H_3_PO_4._ These spectra were used to find more accurately the location of the absorbance peaks of the chromophores. The first peak with a maximum located at *λ*_*m*,1_ = 220 nm is attributed to the presence of a species quoted Ti^*λ*_*m*,1_^ which might also be at the origin of the ^31^P NMR peak observed between *δ* = −6.1 and −6.9 ppm as both the UV-visible absorbance and the ponderate integration values of the ^31^P NMR signal exhibit a dependence *versus* the phosphoric acid concentration (Fig. 9).

A second band can be observed as a shoulder between 260 and 280 nm in the spectra of Fig. 11. In the following part of this paper, the Beer–Lambert law has been used to find the exact position of this second peak (*i.e.*, *λ*_*m*,2_) attributed to the species Ti^*λ*_*m*,2_^ by considering, as stated above, that the contribution to the total absorbance of third species detected by the Malinowski indicator function IND was negligible.

The Beer–Lambert law was expressed by considering two chromophores and a set of wavelengths comprised between 200 and 340 nm:

5

<svg xmlns="http://www.w3.org/2000/svg" version="1.0" width="25.333333pt" height="16.000000pt" viewBox="0 0 25.333333 16.000000" preserveAspectRatio="xMidYMid meet"><metadata>
Created by potrace 1.16, written by Peter Selinger 2001-2019
</metadata><g transform="translate(1.000000,15.000000) scale(0.014583,-0.014583)" fill="currentColor" stroke="none"><path d="M1280 920 l0 -40 -40 0 -40 0 0 -40 0 -40 -40 0 -40 0 0 -40 0 -40 -40 0 -40 0 0 -40 0 -40 -40 0 -40 0 0 -40 0 -40 -40 0 -40 0 0 -40 0 -40 -40 0 -40 0 0 40 0 40 -80 0 -80 0 0 40 0 40 -40 0 -40 0 0 -40 0 -40 -40 0 -40 0 0 -40 0 -40 -40 0 -40 0 0 -80 0 -80 40 0 40 0 0 -40 0 -40 40 0 40 0 0 -40 0 -40 -40 0 -40 0 0 -40 0 -40 -120 0 -120 0 0 40 0 40 40 0 40 0 0 40 0 40 -80 0 -80 0 0 -120 0 -120 160 0 160 0 0 40 0 40 80 0 80 0 0 40 0 40 80 0 80 0 0 -40 0 -40 -40 0 -40 0 0 -40 0 -40 120 0 120 0 0 40 0 40 80 0 80 0 0 40 0 40 40 0 40 0 0 40 0 40 -40 0 -40 0 0 -40 0 -40 -40 0 -40 0 0 80 0 80 40 0 40 0 0 40 0 40 40 0 40 0 0 80 0 80 40 0 40 0 0 40 0 40 40 0 40 0 0 120 0 120 40 0 40 0 0 40 0 40 -80 0 -80 0 0 -40z m-80 -240 l0 -40 -40 0 -40 0 0 -80 0 -80 -40 0 -40 0 0 -40 0 -40 -40 0 -40 0 0 -40 0 -40 -40 0 -40 0 0 -80 0 -80 -40 0 -40 0 0 120 0 120 -40 0 -40 0 0 -80 0 -80 -80 0 -80 0 0 40 0 40 -40 0 -40 0 0 40 0 40 40 0 40 0 0 40 0 40 120 0 120 0 0 -40 0 -40 40 0 40 0 0 40 0 40 40 0 40 0 0 40 0 40 40 0 40 0 0 40 0 40 40 0 40 0 0 40 0 40 40 0 40 0 0 -40z"/></g></svg>

_(*n*)_ = *ε*_Ti^*λm*,1^_[Ti^*λ*_*m*,1_^]_(*n*)_ + *ε*_Ti^*λ*m,2^_[Ti^*λ*_*m*,2_^]_(*n*)_

where _(*n*)_ corresponds to the vector of the absorbance measured between 200 and 340 nm for a given set of experimental condition (*n*) (titanium(iv) and phosphoric acid concentrations as reported for (*n*) = 1 to 4 in Fig. 10). *ε*_Ti^*λ*_*m*,1_^_ and *ε*_Ti^*λ*_*m*,2_^_ denote the vectors of the molar extinction coefficients of the species Ti^*λ*_*m*,1_^ and Ti^*λ*_*m*,2_^ between 200 and 340 nm.

For the set of experimental condition *n* = 1 ([Ti(iv)] = 0.3 mmol L^−1^ and [H_3_PO_4_] = 7.7 mol L^−1^), Fig. 11 shows that the contribution of Ti^*λ*_*m*,2_^ was negligible. Therefore, under these experimental conditions, the contribution of Ti^*λ*_*m*,2_^ in the expression of 𝒜_(1)_ can be neglected as well. The molar extinction coefficient between 200 and 340 nm for Ti^*λ*_*m*,2_^ can thus be expressed as:6
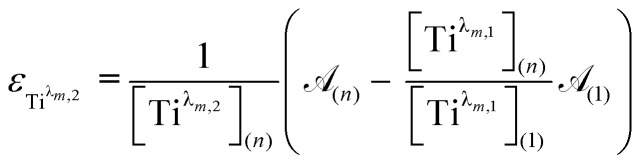


To get information about the peak position Ti^*λ*_*m*,2_^, it is therefore necessary to evaluate the ratio of the concentrations of the first species Ti^*λ*_*m*,1_^ between the two different experimental conditions (*n*) and (1). This ratio may be expressed in eqn (7b) by using the expression of the Beer–Lambert law at *λ*_*m*,1_ = 220 nm [eqn (7a)]:7a
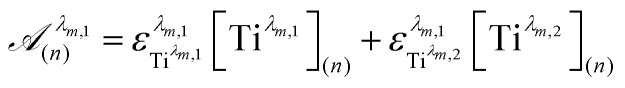
7b
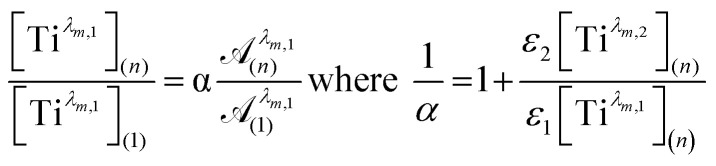


The coefficient *α* corresponds to the contribution of Ti^*λ*_*m*,2_^ in the absorbance at 220 nm under the experimental condition (*n*).

By combining eqn (7a) and (7b), the following expression of the vector Π, which is proportional to *ε*_Ti^*λm*,2^_, can be deduced:8
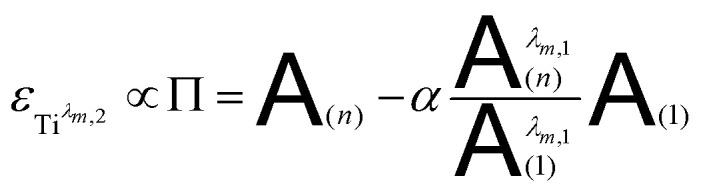


**Fig. 12:**
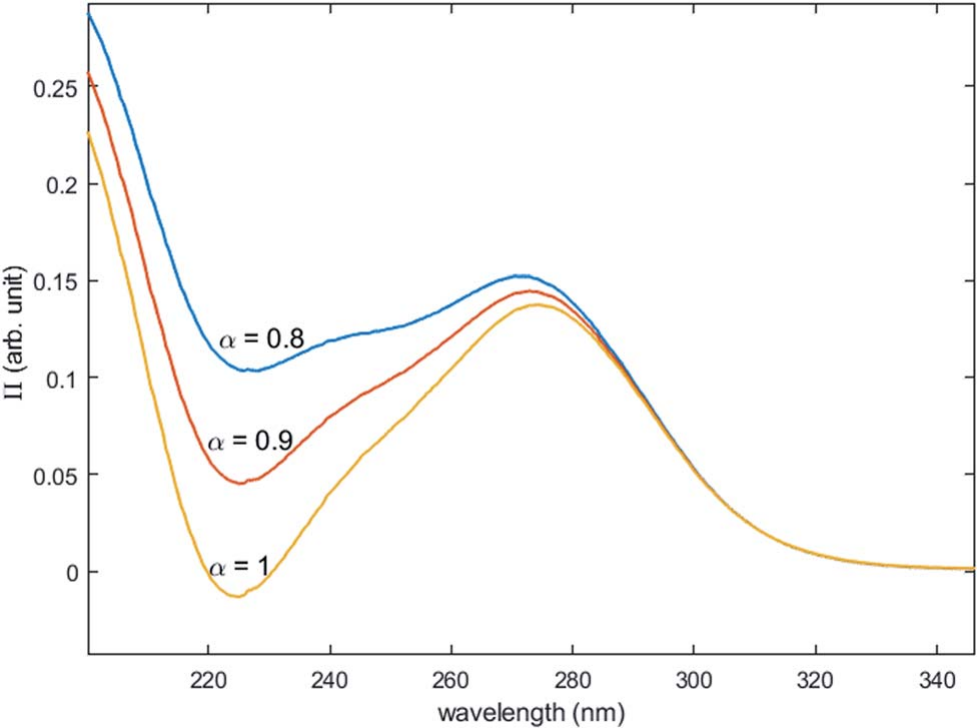
Molar extinction coefficient of Ti^*λ*_*m*,2_^ calculated for *α* = 0.8, 0.9 and 1 by [eqn (9)].

Fig. 12 shows the variation of Π for the species Ti^*λ*_*m*,2_^ at three hypothetic values of *α* as a function of the wavelength (experimental condition (*n*) = 4, *i.e.* 0.3 mmol L^−1^ Ti(iv) and 2 mol L^−1^ H_3_PO_4_; this experimental condition was chosen because the spectra recorded under the experimental conditions corresponding to (*n*) = 1 and (*n*) = 4 show a significant difference between 260–280 nm).

A maximum of extinction coefficient is clearly observed at 270 nm and can be attributed to the second titanium(iv) complex Ti^*λ*_*m*,2_^ present in solution. Fig. 11 shows that the concentration of Ti^*λ*_*m*,2_^ decreases when phosphoric acid concentration is increased (see region 270–320 nm for the conditions (1) to (4) in Fig. 11).

Finally, it can be expected that the following reaction takes place in solution when increasing phosphoric acid concentration from 2 to 7 mol L^−1^:9
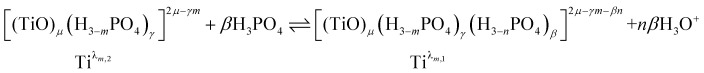
where 1≤ *μ*, 0≤ *m* ≤3, 0≤ *n* ≤3, 1≤ *γ* ≤4*μ* and 1≤ *β* ≤4*μ* are integers. As previously stated, polytitanate formation cannot be excluded as the literature of titanium chemistry reports the ability of titanium(iv) to polymerize.^29^

**Fig. 13:**
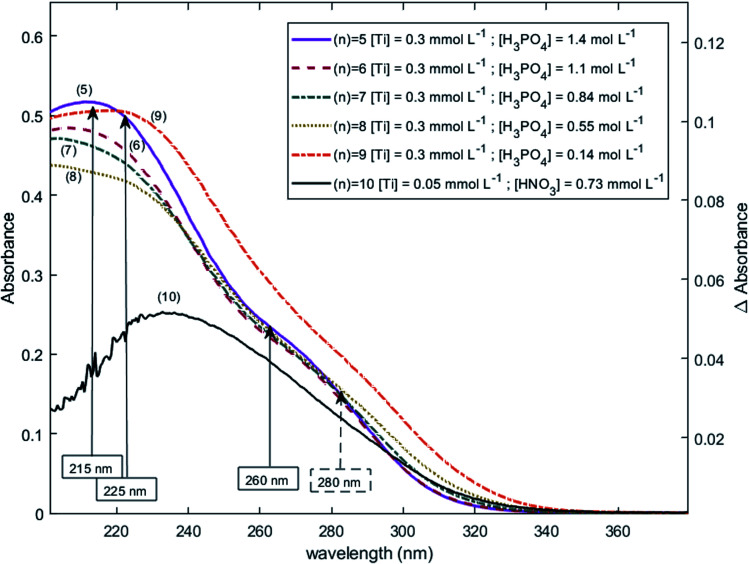
UV-vis spectra of solutions with 0.3 mmol L^−1^ Ti(iv) and a phosphoric acid concentration of 0.1–1.4 mol L^−1^ after baseline correction (2 mm light path cuvette).

The two chromophores Ti^*λ*_*m*,1_^ and Ti^*λ*_*m*,2_^ are still visible in Fig. 13 for phosphoric acid concentration ranging between 0.8 and 1.4 mol L^−1^. For instance, two bands located at 220 nm and 260 nm can be clearly observed in the spectrum recorded for [H_3_PO_4_] = 1.4 mol L^−1^ (curve (5) in Fig. 13). However, another peak corresponding to another chromophore can be observed at 225 nm when phosphoric concentration was lower than 0.5 mol L^−1^ (curves (8) and (9) in Fig. 13). The absorbance at this wavelength increases when phosphoric acid concentration is decreased from 0.5 to 0.14 mol L^−1^. Such an observation suggests that another chromophore, Ti^*λ*_*m*,3_^, exists at low concentration of H_3_PO_4_.

It is interesting to point out that the peak corresponding to this third chromophore Ti^*λ*_*m*,3_^ in the spectra of titanium(iv) in phosphoric acid and the peak observed in the spectrum of titanium(iv) in diluted nitric acid attributed to free titanium(iv) [Fig. 13, black curve (10)] are located at similar wavelength and exhibit the same shape. The shift in wavelength (225 nm against 240 nm) may be attributed to the presence of the absorbance peak of Ti^*λ*_*m*,2_^. Thus, Ti^*λ*_*m*,3_^ can be identified to free titanium(iv).

The absorbance at 310 nm for 0.3 mmol L^−1^ Ti(iv) in 1.4 mol L^−1^ H_3_PO_4_ (Fig. 13, curve (5)) was very low because the concentration of Ti^*λ*_*m*,2_^ was low. A decrease of phosphoric acid concentration from 1.1 to 0.1 mol L^−1^ is responsible for an increase of the absorbance at 310 nm of 0.05 AU, while the absorbance at 260 nm increases of 0.08 AU. The extinction coefficient calculated with eqn (8) shows an absorbance at 260 nm almost 5 times greater than the absorbance at 310 nm (Fig. 13). The presence of a third species is therefore obviously responsible for the increase of absorbance at 310 nm when phosphoric acid concentration decreases. Along with the position of the absorbance peak, this second observation also supports the presence of the free titanium(iv) [(TiO)_*μ*_]^2*μ*^ in solution as Fig. 1b shows also a significant absorbance at this wavelength.

In summary, the variation of the speciation of titanium(iv) from non-complexing aqueous solution to concentrated solution of H_3_PO_4_ can be explained by considering the two following titanium orthophosphate complexes in addition to free titanium(iv), Ti^*λ*_*m*,3_^, corresponding to [(TiO)_*μ*_]^2*μ*^.-Ti^*λ*_*m*,2_^ as [(TiO)_*μ*_(H_3−*m*_PO_4_)_*γ*_]^2*μ*−*γm*^-Ti^*λ*_*m*,1_^ as [(TiO)_*μ*_(H_3−*m*_PO_4_)_*γ*_(H_3−*n*_PO_4_)_*β*_]^2*μ*−*γm*−*βn*^

These complexes are formed in phosphoric acid according to the following reactions depending on phosphoric acid concentration:10
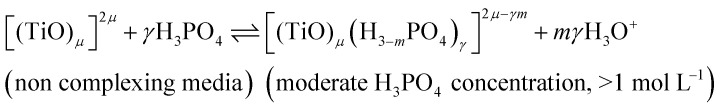
11
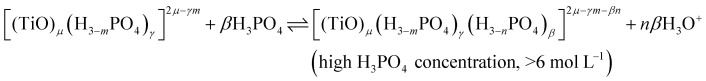
where 1≤ *μ*, 0≤ *m* ≤3 and 1≤ *γ* ≤4*μ* are integers.

In these equilibria, the size *μ* of the complex is kept constant for simplification, but it might evolve with the phosphoric acid concentration.

## 4. Conclusion

The use of UV-visible spectroscopy and molecular modeling techniques was found to be relevant to reinvestigate the speciation of titanium in non-complexing acidic solutions. Indeed, mononuclear species such as titanyl ion or titanium dihydroxide which were considered in previous studies to be predominant in non complexing acidic media were found not to be sufficient to correctly simulate UV-visible spectra by DFT calculations. On the opposite, taking into account the existence of small polytitanate clusters as dimeric or trimeric structures lead to a satisfactory match between experimental and DFT calculated UV-visible spectra. However, in spite of such a good fitting, the determination of the exact nature of the observed polytitanates (*i.e.*, size and hydrolysis state) was not possible due to the multiplicity and similarity of corresponding structures.

UV-visible spectroscopy coupled with chemometric analyses and ^31^P NMR spectroscopy were also successfully used to qualitatively investigate the speciation of titanium(iv) in a large range of phosphoric acid concentrations (*i.e.*, 0.1 to 11.6 mol L^−1^). Thus, it is concluded that titanium(iv) is present as mononuclear (*μ* = 1) or polynuclear (*μ* >1) free cation(s), [(TiO)_*μ*_]^2*μ*^, in diluted H_3_PO_4_ solutions (typically <0.1 mol L^−1^) and then progressively forms orthophosphate complexes as the concentration of phosphoric acid is increased, namely [(TiO)_*μ*_(H_3−*m*_PO_4_)_*γ*_]^2*μ*−*γm*^ and [(TiO)_*μ*_(H_3−*m*_PO_4_)_*γ*_(H_3−*n*_PO_4_)_*β*_]^2*μ*−*γm*−*βn*^ in moderate (typically >1 mol L^−1^) and concentrated (typically >6 mol L^−1^) H_3_PO_4_ solutions, respectively.

A further step in the study of the speciation of titanium(iv) in H_3_PO_4_ solution might be to perform TD-DFT calculation as the one reported above for diluted HNO_3_ solutions (non-complexing media). However, due to the large number of possible structures (*i.e.*, stoichiometry or polytitanate size) and necessary long computing time, such calculations were beyond the scope of the present paper.

## Author contribution

This work was conceptualized by L. M., A. C., G. C. and H. H. The access to the calculation resources was made possible by A. M. The investigation process was performed by L. M., A. M. and S. L. The original draft of the manuscript was written by L. M., A. C. and G. C. All the authors contributed to edit and review the final version of the manuscript. G. C., H. H. and A. C. were involved in the funding acquisition and supervision of the project.

## Conflicts of interest

There are no conflicts to declare.

## Supplementary Material

RA-011-D1RA04284J-s001
