# Supplementary material for: New insights for titanium(iv) speciation in acidic media based on UV-visible and 31P NMR spectroscopies and molecular modeling
Source: RSC Adv. 2021 Aug 9;11(43):27059–73. doi: 10.1039/d1ra04284j (PMC9037697; doi:10.1039/d1ra04284j)
Supplement: RA-011-D1RA04284J-s001 [file RA-011-D1RA04284J-s001.pdf]

Electronic Supplementary Material (ESI) for RSC Advances.
This journal is © The Royal Society of Chemistry 2021

Electronic Supplementary Information

New insights for titanium(IV) speciation in acidic media based on UV-visible and ^{31}P NMR spectroscopies and molecular modeling

Lucas Mangold [a,b], Hubert Halleux[b], Sébastien Leclerc [c], Aurélien Moncomble [d], Gérard Cote [e], Alexandre Chagnes [a].

[a] Université de Lorraine, CNRS, GeoRessources, F-54000 Nancy, France

[b] Prayon, rue Joseph Wauters 144 à, B-4480 Engis, Belgium

[c] Université de Lorraine, CNRS, LEMTA, F-54000 Nancy, France

[d] Univ. Lille, CNRS, UMR 8516 - LASIRE - Laboratoire Avancé de Spectroscopie pour les Interactions la Réactivité et l'Environnement, F-59000 Lille, France

[e] PSL Research University, Chimie ParisTech, CNRS, Institut de Recherche de Chimie-Paris (IRCP) F-75005 Paris, France

Cartesian coordinates of the atoms for the different optimized geometries. Excitation energies and associated oscillator strengths.

Table of content

Ground state optimized geometries – Cartesian coordinates (Angstrom) 3
TiO^{2+} 3
$[Ti(OH)_2]^{2+}$ *(cis)* 3
$[Ti(OH)_2]^{2+}$ *(trans)* 4
$[Ti(OH)_3]^{+}$ 4
$[TiOH]^{3+}$ 5
$[Ti_2(\mu\text{-}O(OH))]^{5+}$ 6
$[Ti_2(\mu\text{-}O(OH))(OH)]^{5+}$ *(ax.)* 7
$[Ti_3(\mu\text{-}(O)_3)]^{6+}$ 8
$[Ti_2(\mu\text{-}(O)_2)]^{4+}$ 9
$[Ti_{18}O_{69}H_{72}]^{6+}$ (**Ti_{18}**) 10
Excitation energies and oscillator strengths: 16
TiO^{2+} 16
$[Ti(OH)_2]^{2+}$ *(trans)* 17
$[Ti(OH)_3]^{+}$ 18
$[TiOH]^{3+}$ 19
$[Ti_2(\mu\text{-}O(OH))]^{5+}$ 20
$[Ti_2(\mu\text{-}O(OH))(OH)]^{5+}$ *(ax.)* 22
$[Ti_3(\mu\text{-}(O)_3)]^{6+}$ 24
$[Ti_2(\mu\text{-}O(OH))]^{5+}$ Canonical MOs depiction of two main excitations 26

Ground state optimized geometries – Cartesian coordinates (Angstrom)

TiO^{2+}

**Atom**	*x*	*y*	*z*
**Ti**	-0.012199	-0.018936	-0.264969
**O**	-0.062418	-0.088962	-1.846842
**O**	-1.434043	1.472322	-0.051892
**H**	-1.935020	1.697729	0.740679
**O**	1.494161	1.398573	-0.157262
**H**	2.018772	1.636513	0.616077
**O**	-1.494721	-1.420362	0.102217
**H**	-1.939135	-1.608134	0.937186
**O**	1.409148	-1.499839	0.017504
**H**	1.944242	-1.660158	0.803552
**O**	0.098774	0.158868	2.012783
**H**	0.084443	0.969312	2.534506
**H**	-1.963802	-1.881569	-0.602722
**H**	1.786309	-2.001392	-0.714356
**H**	0.128855	-0.561616	2.652394
**H**	1.915482	1.772248	-0.939614
**H**	-1.858977	1.888869	-0.810453

$[Ti(OH)_2]^{2+}$ *(cis)*

**Atom**	*x*	*y*	*z*
**Ti**	-0.003079	-0.282039	-0.019001
**O**	0.014542	1.139226	1.538801
**H**	0.015676	0.927889	2.479791
**O**	-0.009539	-1.177787	-1.486648
**H**	-0.009027	-1.383272	-2.427453
**O**	2.031656	0.092426	-0.099163
**H**	2.620131	-0.420872	-0.666664
**O**	0.015752	1.673179	-1.027764
**H**	-0.761034	1.997197	-1.497779
**O**	-0.017469	-1.480008	1.259168

**H**	-0.029139	-2.444279	1.244949
**O**	-2.031071	0.131095	-0.098824
**H**	-2.524984	0.420669	0.678905
**H**	-2.628699	-0.368126	-0.669349
**H**	0.028156	2.096323	1.420164
**H**	2.529998	0.377825	0.677232
**H**	0.795701	1.976453	-1.506337

$[Ti(OH)_2]^{2+}$ *(trans)*

**Atom**	*x*	*y*	*z*
**Ti**	0.000278	0.007113	-0.000012
**O**	1.418256	-1.475295	-0.000075
**H**	1.823185	-1.854407	0.788779
**O**	-0.001030	-0.025221	1.761786
**H**	-0.009447	-0.162294	2.712304
**O**	1.483506	1.421938	-0.000301
**H**	1.872731	1.817836	-0.789015
**O**	-1.404642	1.500521	0.000097
**H**	-1.785318	1.902661	-0.788727
**O**	-0.001536	-0.025540	-1.761912
**H**	-0.012210	-0.162872	-2.712333
**O**	-1.492890	-1.400113	0.000334
**H**	-1.908899	-1.766687	0.789213
**H**	1.823080	-1.854537	-0.788923
**H**	-1.785996	1.902012	0.789637
**H**	1.872670	1.818196	0.788266
**H**	-1.909219	-1.766694	-0.788378

$[Ti(OH)_3]^{+}$

**Atom**	*x*	*y*	*z*
**Ti**	-0.339357	0.000557	0.003006
**O**	1.252321	1.535888	-0.140888
**H**	1.055926	2.455545	0.064870
**O**	-1.088525	-1.605618	0.270847
**O**	1.236772	-0.880595	-1.279524
**H**	1.025835	-1.160585	-2.176096
**O**	-1.067195	1.033770	1.273967
**H**	-1.691023	1.756178	1.157586
**O**	1.267099	-0.668000	1.381513
**O**	-1.106517	0.582118	-1.510869

**H**	-1.727010	0.097477	-2.063134
**H**	-1.714814	-1.852018	0.957668
**H**	1.815385	-1.545362	-0.890537
**H**	1.055752	-1.273103	2.100012
**H**	1.835676	1.521187	-0.907418
**H**	1.858492	0.007925	1.730544

$[TiOH]^{3+}$

**Atom**	*x*	*y*	*z*
**Ti**	0.001773	0.000431	-0.206541
**O**	0.156864	-1.972160	0.030270
**H**	0.213728	-2.624148	-0.685477
**O**	-0.141984	1.978080	0.000570
**H**	-0.175063	2.449586	0.847539
**O**	-0.016275	0.009695	1.886634
**H**	-0.809060	-0.045845	2.438118
**O**	-0.001102	-0.019916	-1.885918
**H**	-0.016560	-0.033852	-2.855994
**O**	2.006529	0.149634	0.122759
**H**	2.620330	-0.590913	0.008514
**O**	-2.006286	-0.148355	0.113536
**H**	-2.614792	0.598293	0.010697
**H**	-0.180313	2.617714	-0.727349
**H**	0.760244	0.065330	2.460647
**H**	2.505539	0.974957	0.032198
**H**	-2.507056	-0.967761	-0.013387
**H**	0.182049	-2.428686	0.885585

$[Ti_2(\mu\text{-}O(OH))]^{5+}$

Atom	*x*	*y*	*z*
**Ti**	1.424311	0.047183	-0.161987
**O**	1.621904	-1.956453	-0.273009
**H**	1.280806	-2.514495	-0.988721
**O**	1.669129	2.040333	-0.118961
**H**	1.297876	2.601783	0.579133
**O**	2.347899	-0.226583	1.629137
**H**	1.858045	-0.291044	2.462546
**O**	-0.081247	-0.179737	-1.320335
**O**	3.373711	0.161111	-0.809346
**O**	-0.007274	0.223645	0.938682
**H**	1.950007	2.604185	-0.856885
**H**	3.298119	-0.294144	1.806281
**H**	4.011266	0.876942	-0.668310
**H**	2.163918	-2.497647	0.321166
**H**	3.821610	-0.552785	-1.288598
**Ti**	-1.527311	0.005054	0.045408
**O**	-1.660423	-1.764716	0.977753
**H**	-1.234192	-1.963074	1.826467
**O**	-2.091363	1.683368	-0.907804
**H**	-2.031690	1.876657	-1.855542
**O**	-2.562024	-1.040252	-1.383536
**H**	-2.283893	-1.814742	-1.894574
**O**	-2.389005	1.006528	1.578234
**H**	-1.897748	1.295537	2.361897
**H**	-3.498205	-0.869039	-1.571765
**H**	-2.366926	-2.411219	0.820854
**H**	-3.341579	1.051032	1.757776
**H**	-2.440661	2.461140	-0.446784
**H**	-0.091226	-0.286257	-2.286722

$[Ti_2(\mu\text{-}O(OH))(OH)]^{5+}$ *(ax.)*

**Atom**	*x*	*y*	*z*
**Ti**	-1.516346	-0.156573	0.004810
**O**	-1.781794	-1.826871	0.191350
**H**	-2.195194	-2.699315	0.244663
**O**	-1.802654	1.903656	-0.344207
**H**	-2.569807	2.440840	-0.107266
**O**	-2.882730	-0.186582	-1.510023
**O**	0.041533	0.059987	1.251399
**O**	-2.734360	0.309899	1.582497
**O**	0.054190	-0.040730	-1.081771
**H**	-1.223681	2.445919	-0.896158
**H**	-2.668799	1.064153	2.184588
**Ti**	1.434978	-0.000918	-0.072044
**O**	1.723492	-1.983576	-0.380316
**O**	1.933665	1.922346	0.405214
**H**	1.538666	2.432880	1.125612
**O**	2.750122	-0.473826	1.523492
**O**	2.722531	0.449859	-1.616287
**H**	2.433567	0.447469	-2.539326
**H**	2.684913	-1.174920	2.185493
**H**	2.529002	-2.464573	-0.142200
**H**	0.079108	0.010759	2.215911
**H**	3.689956	0.451029	-1.598231
**H**	-3.150773	0.570573	-2.047165
**H**	3.409623	0.159996	1.836621
**H**	-3.284841	-0.364006	2.004280
**H**	-3.301087	-0.985469	-1.856412
**H**	1.273775	-2.473870	-1.082859
**H**	2.353709	2.530045	-0.219182

$[Ti_3(\mu\text{-}(O)_3)]^{6+}$

Atom	x	y	z
Ti	-0.597070	2.018171	0.000140
O	0.685573	3.791257	0.000302
O	-0.634387	2.402415	2.026816
O	-1.763867	-1.750541	-2.026741
O	-0.634289	2.402717	-2.026480
O	-2.159066	3.421640	0.000209
O	-3.626279	-1.301836	-0.000174
O	-1.405919	0.529014	0.000010
O	2.398588	-0.651818	-2.026512
Ti	2.046302	-0.491974	0.000008
O	2.940597	-2.489453	-0.000133
O	4.042882	0.158779	0.000096
O	-1.884118	-3.580379	-0.000316
O	0.244817	-1.482023	-0.000108
Ti	-1.449264	-1.526101	-0.000144
O	1.161013	0.953052	0.000105
O	2.398510	-0.652136	2.026515
O	-1.763945	-1.750866	2.026405
H	-1.991083	-4.162853	-0.775159
H	-1.991137	-4.162971	0.774431
H	-4.263734	-2.041072	-0.000247
H	-4.177526	-0.500959	-0.000119
H	-2.610040	3.805556	0.775019
H	-2.610006	3.805669	-0.774566
H	3.899489	-2.672068	-0.000131
H	2.522484	-3.367211	-0.000207
H	4.600929	0.357284	-0.774675
H	1.654779	3.868210	0.000333
H	-0.192182	3.179921	2.419560
H	2.385631	0.036695	2.714836
H	-1.160992	-2.084023	2.714802
H	-1.160890	-2.083586	-2.715171
H	2.850838	-1.423642	-2.419023
H	-0.192066	3.180282	-2.419086
H	4.600897	0.357170	0.774920
H	0.364116	4.712932	0.000362
H	2.385742	0.037120	-2.714726
H	-2.658466	-1.756214	-2.419132

H	-1.224516	2.047046	-2.714634
H	-1.224647	2.046641	2.714888
H	2.850749	-1.424018	2.418924
H	-2.658557	-1.756598	2.418765

$[Ti_2(\mu\text{-}(O)_2)]^{4+}$

Atom	x	y	z
Ti	-1.33513	-0.03952	-0.15243
O	-1.58194	1.95657	-0.46502
H	-1.23849	2.43766	-1.2308
O	-1.53585	-2.06535	0.03828
H	-1.22154	-2.56536	0.80386
O	-2.42925	0.42609	1.58207
H	-2.00481	0.56814	2.43788
O	0.07129	0.08648	-1.25005
O	-3.30908	-0.27498	-0.82829
O	-0.00928	-0.12288	1.10568
H	-1.79842	-2.68664	-0.65549
H	-3.3847	0.52708	1.68076
H	-3.91523	-0.9935	-0.60679
H	-2.20226	2.52185	0.01575
H	-3.70119	0.24362	-1.54365
Ti	1.37587	0.00094	-0.00126
O	1.53492	1.95798	0.53671
H	1.16644	2.33571	1.34694
O	1.66248	-1.94617	-0.52024
H	1.43066	-2.34601	-1.36966
O	2.78384	0.64036	-1.4365
H	2.53599	0.89238	-2.33562
O	2.71007	-0.58848	1.50743
H	2.43414	-0.66682	2.43046
H	3.74766	0.67189	-1.37249
H	2.18194	2.57169	0.1632
H	3.67363	-0.66313	1.47864
H	2.22243	-2.55676	-0.0223

$[Ti_{18}O_{69}H_{72}]^{6+}$ (**Ti_{18}**)

A previously reported titanium oxo-cluster[1] was used in order to build the structure of the species $[Ti_{18}O_{69}H_{72}]^{6+}$. The structure of the pentagonal Ti-O cluster was described in the crystallographic file as part of the supplementary information of the work of Kozma et al.[1]. The organic molecules and free water molecules as well as the chlorides present in the structure were removed and the sulfates groups coordinated to titanium atoms of the cluster were replaced by hydroxides in order to get the starting geometry of a free titanium oxo-cluster containing three stacked pentagonal moiety. This geometry was then optimized geometrically using PBE0 functional and 3-21G basis set and no solvation model. The cartesian coordinates of the optimized structures are given below. A frequency calculation showed that the vibration modes for this geometry were all associated with positive eigenvalues confirming that this geometry is a local minimum. 300 excited states were then computed using the same level of theory (PBE0 and 3-21G without any solvation model). This amount of states allowed to reach an energy of 5.55 eV (~223 nm) for the 300th computed excited state. Therefore, the existence of excited states below 223 nm associated with high oscillator strengths cannot be excluded, leading to an eventual shift of the maximal wavelength of the convoluted spectrum toward smaller wavelengths. However, the use of the above mentioned computational parameters tends to show that the position of the predicted maximum of **Ti_{18}** spectrum by TD-DFT might be around 235-240 nm. In addition, the basis set (3-21G) used for this system is small but the purpose of this calculation was to illustrate a tendency as shown on Figure 8 of the manuscript.

[1] K. Kozma, M. Wang, P. I. Molina, N. P. Martin, Z. Feng and M. Nyman, *Dalton Trans.*, 2019, **48**, 11086–11093.

Atom	x	y	z
O	5.73706	0.28242	-0.19213
H	5.98606	1.25115	-0.54342
H	6.05349	-0.41229	-0.93665
O	1.7884	0.13973	-0.10012
O	-1.86343	-0.09269	0.16057
O	-5.83831	-0.1552	-0.31287
H	-5.98839	1.16138	-0.61031
H	-6.11005	-0.65216	-1.19429
O	5.64836	-2.25168	2.62192
H	6.11825	-3.08808	2.38414
H	6.27806	-1.24769	2.37968
O	6.58772	-1.1483	-2.13518
H	5.66477	-2.00421	-2.68795
H	7.42498	-1.63755	-1.92851
O	6.9817	1.00263	-3.16334
H	7.70181	1.19959	-3.80748
H	6.82054	-0.146	-2.75032
O	6.45327	2.49859	-1.22676
H	7.21648	2.96852	-0.8055
H	6.82269	1.74322	-2.4257
O	6.77419	-0.22696	2.00459
H	7.70709	0.00049	2.21515
H	6.38304	0.06133	1.05848
O	3.4382	0.22199	4.55478
H	3.66509	-0.79325	4.5642
H	3.38384	0.62523	5.45139
O	3.74593	-0.28543	2.01189
O	4.00898	-2.20647	4.25575

H	3.73507	-2.91036	4.89313
H	5.02217	-2.29185	3.85667
O	3.60726	-1.84708	0.2713
O	3.66521	-0.69825	-1.82656
O	3.62503	1.62631	-1.34247
O	3.41956	1.89933	1.00753
O	3.80288	-3.96762	1.69085
O	4.41668	-4.06393	-0.63488
O	4.11293	0.10463	-4.45699
O	3.85517	2.60102	-3.61521
H	4.13416	2.84508	-4.52539
H	4.12166	3.29321	-2.8598
O	4.47045	3.92719	-1.603
H	5.59763	3.18711	-1.39903
H	4.61022	4.90728	-1.59932
O	4.55314	2.48431	3.49567
O	3.31869	4.58634	0.73095
O	-0.83958	0.95182	4.24565
H	-0.63187	-0.08008	4.39419
H	-1.76027	1.20128	4.59126
O	-0.48417	-1.53445	4.28937
H	0.11363	-1.88522	4.99505
H	-1.75666	-1.9202	4.45526
O	1.96541	-5.52649	1.19888
H	2.76964	-4.89888	1.53468
H	2.05994	-6.49133	1.35772
O	-0.01752	-0.04927	2.01422
O	0.26756	-1.88406	0.43371
O	1.76779	-2.35036	2.64986
O	1.66582	1.75223	3.15447
O	1.8693	-3.02279	-1.72109
O	1.58762	0.63977	-3.34178
O	1.42941	3.45916	-0.98658

O	-0.13908	-1.05169	-1.77831
O	-0.33845	1.36094	-1.61863
O	0.02353	1.95278	0.75118
O	-0.05926	-1.44142	-4.30358
H	-0.25323	-2.4155	-3.83171
H	0.13989	-1.47348	-5.26607
O	-0.25233	1.44042	-4.81736
H	-1.10175	1.60685	-5.28848
H	0.53326	1.81427	-5.27874
O	-1.15275	4.06674	-2.26308
H	-0.712	4.58413	-2.97475
H	-2.28549	4.12827	-2.2558
O	-0.34167	4.80291	0.52466
H	-1.32071	4.99169	0.9411
H	0.12317	5.5986	0.16736
O	0.28355	3.80529	2.73394
H	0.18434	4.31868	1.82899
H	1.06653	4.09328	3.25791
O	-1.56879	-2.69047	2.19172
O	-1.74814	-3.16586	-0.79167
O	-2.15104	3.10125	-0.19019
O	-1.96048	2.46683	2.55933
O	-2.86879	-2.11253	4.32733
O	-3.49654	1.29852	4.53345
O	-4.64588	-3.46089	2.43515
H	-5.90853	-2.41953	1.81043
H	-4.86083	-4.02805	3.21857
O	-6.53188	-1.68107	1.50105
H	-6.21632	-0.75133	0.5309
H	-7.43637	-1.76972	1.88274
O	-3.44608	-0.31415	2.27301
O	-3.80956	-2.02653	0.58436
O	-3.62221	-1.24646	-1.67267

O	-3.65557	1.15622	-1.39944
O	-4.06822	1.58597	0.93335
O	-5.37738	-3.36375	-1.64725
H	-5.97833	-2.56746	-2.06081
H	-5.89122	-4.14156	-1.33357
O	-4.73898	-0.92515	-4.14021
O	-5.08575	1.49567	-3.35672
O	-2.06116	0.33296	-3.57979
O	-4.5853	4.19671	0.78727
H	-3.40348	4.7203	1.32844
H	-5.1969	4.91523	0.48807
O	-2.43992	4.89306	1.81506
H	-2.08691	3.47976	2.28494
H	-2.48783	5.57265	2.53399
O	-4.88414	2.89646	2.85406
H	-5.332	3.25756	3.6536
H	-4.95939	3.49865	1.94117
O	-5.93368	2.1913	-1.08844
H	-5.55534	1.88733	-2.49402
H	-6.77048	2.70068	-0.9507
O	-6.48622	-1.37053	-2.52777
H	-5.77205	-1.19462	-3.42396
H	-7.44634	-1.37359	-2.76859
O	-3.60919	3.99935	-2.05254
Ti	3.53713	0.16187	0.01687
Ti	-0.00815	0.06833	-0.06977
Ti	-3.57555	-0.14446	0.09831
Ti	3.52085	-2.27354	2.11734
Ti	3.50889	-2.52684	-1.4496
Ti	3.33039	0.74348	-3.01472
Ti	3.08366	3.18294	-0.33693
Ti	3.4101	1.33352	2.83106
Ti	0.10163	-1.82245	2.28189

Ti	0.04293	-2.87305	-1.13426
Ti	-0.15864	0.18731	-3.12933
Ti	-0.33281	2.99568	-0.77669
Ti	-0.02144	1.76533	2.60754
Ti	-3.21586	-2.1554	2.37245
Ti	-3.4743	-3.00614	-1.0273
Ti	-3.63985	0.10736	-2.94344
Ti	-3.80208	2.82012	-0.54597
Ti	-3.51436	1.47856	2.72217
O	-2.96025	-3.86508	-2.7768
H	-3.82007	-5.50793	0.19199
H	-4.37303	-4.21718	1.17867
O	-4.01731	-4.54438	0.23323
H	-1.89237	-3.79776	-2.99543
O	-0.50766	-3.51411	-3.05149
H	0.03064	-4.28505	-3.36505
H	-0.59342	-4.93907	0.08504
O	0.27276	-4.59345	-0.23731
H	1.17762	-5.10658	0.43211
O	4.84671	-2.71922	-2.90046
H	4.83633	-4.84768	-1.05641
H	4.97917	-3.32478	-3.66402
H	4.24256	-4.12884	0.52243
H	-3.90918	1.37284	5.42355
H	-3.40627	-2.03953	5.1483
H	-3.53212	-4.21858	-3.49479
H	-4.62654	-1.34856	-5.02037
H	-5.65103	1.4656	-4.16269
H	-4.19315	4.37875	-2.74859
H	4.70787	-0.04553	-5.21935
H	3.82555	5.19653	1.30837
H	5.3179	2.85246	3.98646

Excitation energies and oscillator strengths:

TiO^{2+}

State	Energy	Wavelength	Oscillator strength
1:	5.3175 eV	233.16 nm	f=0.0000
2:	5.3880 eV	230.11 nm	f=0.0021
3:	5.4480 eV	227.58 nm	f=0.0002
4:	5.9672 eV	207.78 nm	f=0.0000
5:	6.0041 eV	206.50 nm	f=0.0017
6:	6.0787 eV	203.97 nm	f=0.0022
7:	6.3367 eV	195.66 nm	f=0.0000
8:	6.4308 eV	192.80 nm	f=0.0002
9:	6.4584 eV	191.97 nm	f=0.0001
10:	6.5114 eV	190.41 nm	f=0.0003
11:	6.8292 eV	181.55 nm	f=0.0860
12:	7.0126 eV	176.80 nm	f=0.0118
13:	7.0307 eV	176.35 nm	f=0.0014
14:	7.0450 eV	175.99 nm	f=0.0784
15:	7.0790 eV	175.14 nm	f=0.0079
16:	7.3776 eV	168.06 nm	f=0.0073
17:	7.4482 eV	166.46 nm	f=0.0028
18:	7.4870 eV	165.60 nm	f=0.0419
19:	7.5294 eV	164.67 nm	f=0.0000
20:	7.7221 eV	160.56 nm	f=0.0092

$[Ti(OH)_2]^{2+}$ *(cis)*

State	Energy	Wavelength	Oscillator strength
1:	5.0278 eV	246.60 nm	f=0.0004
2:	5.1843 eV	239.15 nm	f=0.0002
3:	5.2704 eV	235.25 nm	f=0.0006
4:	5.4642 eV	226.90 nm	f=0.0010
5:	5.5261 eV	224.36 nm	f=0.0004
6:	5.8975 eV	210.23 nm	f=0.0008
7:	5.9055 eV	209.95 nm	f=0.0074
8:	6.0408 eV	205.24 nm	f=0.0000
9:	6.0931 eV	203.48 nm	f=0.0121
10:	6.1825 eV	200.54 nm	f=0.0001
11:	6.3339 eV	195.75 nm	f=0.0638
12:	6.3514 eV	195.21 nm	f=0.0010
13:	6.5645 eV	188.87 nm	f=0.0376
14:	6.6387 eV	186.76 nm	f=0.0074

15:	6.6806 eV	185.59 nm	f=0.0210
16:	6.7676 eV	183.20 nm	f=0.0021
17:	6.7966 eV	182.42 nm	f=0.0132
18:	6.8524 eV	180.93 nm	f=0.0006
19:	6.8645 eV	180.62 nm	f=0.0096
20:	6.8943 eV	179.84 nm	f=0.0040
21:	6.9313 eV	178.88 nm	f=0.0102
22:	7.0824 eV	175.06 nm	f=0.0261
23:	7.1726 eV	172.86 nm	f=0.0120
24:	7.2556 eV	170.88 nm	f=0.0096
25:	7.3061 eV	169.70 nm	f=0.0035
26:	7.4887 eV	165.56 nm	f=0.0262
27:	7.6407 eV	162.27 nm	f=0.0007
28:	7.7239 eV	160.52 nm	f=0.0220

$[Ti(OH)_2]^{2+}$ *(trans)*

State	Energy	Wavelength	Oscillator strength
1:	4.6037 eV	269.31 nm	f=0.0026
2:	4.6178 eV	268.49 nm	f=0.0025
3:	4.9825 eV	248.84 nm	f=0.0000
4:	5.1555 eV	240.49 nm	f=0.0000
5:	5.1583 eV	240.36 nm	f=0.0000
6:	5.4072 eV	229.30 nm	f=0.0000
7:	5.4133 eV	229.04 nm	f=0.0000
8:	5.7958 eV	213.92 nm	f=0.0000
9:	5.8049 eV	213.59 nm	f=0.0000
10:	6.0767 eV	204.03 nm	f=0.0000
11:	6.0847 eV	203.76 nm	f=0.0000
12:	6.2452 eV	198.53 nm	f=0.0296
13:	6.2636 eV	197.94 nm	f=0.0284
14:	6.3258 eV	196.00 nm	f=0.0000
15:	6.3309 eV	195.84 nm	f=0.0001
16:	6.3613 eV	194.90 nm	f=0.0793
17:	6.3766 eV	194.44 nm	f=0.0800
18:	6.7998 eV	182.33 nm	f=0.1045
19:	6.8120 eV	182.01 nm	f=0.0013
20:	6.8205 eV	181.78 nm	f=0.0331
21:	6.8851 eV	180.08 nm	f=0.0000
22:	7.0893 eV	174.89 nm	f=0.4163
23:	7.1122 eV	174.33 nm	f=0.0001
24:	7.1191 eV	174.16 nm	f=0.0274
25:	7.1660 eV	173.02 nm	f=0.0000
26:	7.2297 eV	171.49 nm	f=0.0000
27:	7.4861 eV	165.62 nm	f=0.0241
28:	7.4937 eV	165.45 nm	f=0.0231

29:	7.7356 eV	160.28 nm	f=0.0001
30:	7.7395 eV	160.20 nm	f=0.0000

$[Ti(OH)_3]^+$

State	Energy	Wavelength	Oscillator strength
1:	5.4190 eV	228.80 nm	f=0.0000
2:	5.4259 eV	228.50 nm	f=0.0000
3:	5.4346 eV	228.14 nm	f=0.0000
4:	5.5079 eV	225.10 nm	f=0.0002
5:	5.5141 eV	224.85 nm	f=0.0006
6:	5.5270 eV	224.32 nm	f=0.0006
7:	5.6024 eV	221.31 nm	f=0.0021
8:	5.8695 eV	211.24 nm	f=0.0401
9:	5.8728 eV	211.12 nm	f=0.0399
10:	6.2156 eV	199.47 nm	f=0.0036
11:	6.2217 eV	199.28 nm	f=0.0033
12:	6.2543 eV	198.24 nm	f=0.0025
13:	6.3865 eV	194.14 nm	f=0.0001
14:	6.4680 eV	191.69 nm	f=0.0028
15:	6.4733 eV	191.53 nm	f=0.0022
16:	6.5618 eV	188.95 nm	f=0.0541
17:	6.5644 eV	188.87 nm	f=0.0549
18:	6.8880 eV	180.00 nm	f=0.0067
19:	7.0523 eV	175.81 nm	f=0.0004
20:	7.1074 eV	174.44 nm	f=0.0003
21:	7.1186 eV	174.17 nm	f=0.0004
22:	7.3522 eV	168.64 nm	f=0.0012
23:	7.3693 eV	168.24 nm	f=0.0009
24:	7.4175 eV	167.15 nm	f=0.0075
25:	7.4282 eV	166.91 nm	f=0.0004
26:	7.4438 eV	166.56 nm	f=0.0017
27:	7.4545 eV	166.32 nm	f=0.0023
29:	7.5221 eV	164.83 nm	f=0.0036
30:	7.5632 eV	163.93 nm	f=0.0065
31:	7.7146 eV	160.71 nm	f=0.0704
32:	7.7235 eV	160.53 nm	f=0.0715

$[TiOH]^{3+}$

State	Energy	Wavelength	Oscillator strength
1:	4.5622 eV	271.76 nm	f=0.0002
2:	4.9696 eV	249.48 nm	f=0.0003
3:	5.0163 eV	247.16 nm	f=0.0000
4:	5.2641 eV	235.53 nm	f=0.0000
5:	5.3301 eV	232.61 nm	f=0.0001
6:	5.4493 eV	227.52 nm	f=0.0000
7:	5.6198 eV	220.62 nm	f=0.0000
8:	5.7594 eV	215.27 nm	f=0.0713
9:	5.9902 eV	206.98 nm	f=0.0644
10:	5.9971 eV	206.74 nm	f=0.0718
11:	6.0117 eV	206.24 nm	f=0.0003
12:	6.0364 eV	205.39 nm	f=0.0063
13:	6.1319 eV	202.20 nm	f=0.0002
14:	6.2265 eV	199.12 nm	f=0.0269
15:	6.2387 eV	198.74 nm	f=0.0370
16:	6.3400 eV	195.56 nm	f=0.0040
17:	6.4038 eV	193.61 nm	f=0.0008
18:	6.5219 eV	190.10 nm	f=0.0000
19:	6.5371 eV	189.66 nm	f=0.0057
20:	6.6556 eV	186.29 nm	f=0.0022
21:	6.7496 eV	183.69 nm	f=0.0289
22:	7.1236 eV	174.05 nm	f=0.0081
23:	7.4133 eV	167.24 nm	f=0.0109
24:	7.4192 eV	167.11 nm	f=0.0116
25:	7.4913 eV	165.50 nm	f=0.0001
26:	7.6215 eV	162.68 nm	f=0.0258
27:	7.6632 eV	161.79 nm	f=0.0000

$[Ti_2(\mu\text{-}O(OH))]^{5+}$

State	Energy	Wavelength	Oscillator strength
1:	4.4578 eV	278.13 nm	f=0.0311
2:	4.6131 eV	268.76 nm	f=0.0082
3:	4.6240 eV	268.13 nm	f=0.0063
4:	4.7196 eV	262.70 nm	f=0.0013
5:	4.7952 eV	258.56 nm	f=0.0011
6:	4.8311 eV	256.64 nm	f=0.0080
7:	4.8817 eV	253.98 nm	f=0.0037
8:	4.9280 eV	251.59 nm	f=0.0015
9:	4.9524 eV	250.35 nm	f=0.0111
10:	4.9647 eV	249.73 nm	f=0.0031
11:	5.0069 eV	247.63 nm	f=0.0033
12:	5.0664 eV	244.72 nm	f=0.0101
13:	5.0804 eV	244.04 nm	f=0.0023
14:	5.1130 eV	242.49 nm	f=0.0198
15:	5.1348 eV	241.46 nm	f=0.0046
16:	5.1795 eV	239.38 nm	f=0.0089
17:	5.2126 eV	237.86 nm	f=0.0087
18:	5.2332 eV	236.92 nm	f=0.0041
19:	5.2701 eV	235.26 nm	f=0.0400
20:	5.2931 eV	234.24 nm	f=0.0131
21:	5.3016 eV	233.86 nm	f=0.0173
22:	5.3250 eV	232.84 nm	f=0.0272
23:	5.3656 eV	231.07 nm	f=0.0028
24:	5.3718 eV	230.80 nm	f=0.0472
25:	5.4194 eV	228.78 nm	f=0.0356
26:	5.4426 eV	227.80 nm	f=0.0022
27:	5.4619 eV	227.00 nm	f=0.0906
28:	5.5518 eV	223.32 nm	f=0.0128
29:	5.5797 eV	222.21 nm	f=0.0314
30:	5.6040 eV	221.24 nm	f=0.0115
31:	5.6405 eV	219.81 nm	f=0.0124
32:	5.7183 eV	216.82 nm	f=0.0081
33:	5.7397 eV	216.01 nm	f=0.0050
34:	5.7551 eV	215.43 nm	f=0.0070
35:	5.7778 eV	214.59 nm	f=0.0062
36:	5.7938 eV	213.99 nm	f=0.0183
37:	5.8143 eV	213.24 nm	f=0.0013
38:	5.8377 eV	212.39 nm	f=0.0044
39:	5.8608 eV	211.55 nm	f=0.0029
40:	5.8674 eV	211.31 nm	f=0.0148
41:	5.9320 eV	209.01 nm	f=0.0152
42:	5.9700 eV	207.68 nm	f=0.0155
43:	6.0090 eV	206.33 nm	f=0.0043

44:	6.0351	eV	205.44	nm	f=0.0025
45:	6.0831	eV	203.82	nm	f=0.0047
46:	6.1040	eV	203.12	nm	f=0.0037
47:	6.1178	eV	202.66	nm	f=0.0037
48:	6.1414	eV	201.88	nm	f=0.0002
49:	6.1594	eV	201.29	nm	f=0.0295
50:	6.2107	eV	199.63	nm	f=0.0145
51:	6.2230	eV	199.24	nm	f=0.0011
52:	6.2705	eV	197.73	nm	f=0.0187
53:	6.2868	eV	197.21	nm	f=0.0058
54:	6.3060	eV	196.61	nm	f=0.0048
55:	6.4092	eV	193.45	nm	f=0.0145
56:	6.4290	eV	192.85	nm	f=0.0083
57:	6.4429	eV	192.43	nm	f=0.0033
58:	6.5062	eV	190.56	nm	f=0.0038
59:	6.5531	eV	189.20	nm	f=0.0043
60:	6.5959	eV	187.97	nm	f=0.0046
61:	6.6186	eV	187.33	nm	f=0.0047
62:	6.6259	eV	187.12	nm	f=0.0024
63:	6.7072	eV	184.85	nm	f=0.0025
64:	6.7263	eV	184.33	nm	f=0.0007
65:	6.7697	eV	183.14	nm	f=0.0012
66:	6.7801	eV	182.86	nm	f=0.0055
67:	6.7916	eV	182.56	nm	f=0.0070
68:	6.8213	eV	181.76	nm	f=0.0006
69:	6.8452	eV	181.13	nm	f=0.0043
70:	6.8710	eV	180.45	nm	f=0.0142
71:	6.8953	eV	179.81	nm	f=0.0043
72:	6.9475	eV	178.46	nm	f=0.0182
73:	6.9653	eV	178.00	nm	f=0.0038
74:	6.9956	eV	177.23	nm	f=0.0014
75:	7.0149	eV	176.74	nm	f=0.0084
76:	7.0323	eV	176.31	nm	f=0.0078
77:	7.0663	eV	175.46	nm	f=0.0047
78:	7.1017	eV	174.58	nm	f=0.0015
79:	7.1167	eV	174.22	nm	f=0.0077
80:	7.1405	eV	173.63	nm	f=0.0007

[Ti_2(μ-O(OH))(OH)]$^{5+}$ *(ax.)*

State	Energy	Wavelength	Oscillator strength
1:	4.8102 eV	257.75 nm	f=0.0006
2:	4.9100 eV	252.51 nm	f=0.0101
3:	4.9775 eV	249.09 nm	f=0.0029
4:	5.0695 eV	244.57 nm	f=0.0059
5:	5.1418 eV	241.13 nm	f=0.0006
6:	5.2609 eV	235.67 nm	f=0.0013
7:	5.2877 eV	234.48 nm	f=0.0029
8:	5.3396 eV	232.20 nm	f=0.0016
9:	5.4057 eV	229.36 nm	f=0.0049
10:	5.4257 eV	228.51 nm	f=0.0023
11:	5.4644 eV	226.89 nm	f=0.0058
12:	5.4857 eV	226.01 nm	f=0.0016
13:	5.5354 eV	223.98 nm	f=0.0089
14:	5.5861 eV	221.95 nm	f=0.0070
15:	5.6558 eV	219.22 nm	f=0.0277
16:	5.6694 eV	218.69 nm	f=0.0073
17:	5.6814 eV	218.23 nm	f=0.0309
18:	5.6898 eV	217.90 nm	f=0.0034
19:	5.7499 eV	215.63 nm	f=0.0694
20:	5.7882 eV	214.20 nm	f=0.0118
21:	5.8704 eV	211.20 nm	f=0.1064
22:	5.9268 eV	209.19 nm	f=0.0088
23:	5.9430 eV	208.62 nm	f=0.0166
24:	5.9651 eV	207.85 nm	f=0.0109
25:	5.9977 eV	206.72 nm	f=0.0234
26:	6.0378 eV	205.35 nm	f=0.0210
27:	6.0686 eV	204.31 nm	f=0.0057
28:	6.0896 eV	203.60 nm	f=0.0089
29:	6.1090 eV	202.95 nm	f=0.0053
30:	6.1278 eV	202.33 nm	f=0.0051
31:	6.1496 eV	201.61 nm	f=0.0047
32:	6.2013 eV	199.93 nm	f=0.0082
33:	6.2222 eV	199.26 nm	f=0.0122
34:	6.2737 eV	197.63 nm	f=0.0039
35:	6.3160 eV	196.30 nm	f=0.0022
36:	6.3334 eV	195.76 nm	f=0.0119
37:	6.3622 eV	194.88 nm	f=0.0066
38:	6.3653 eV	194.78 nm	f=0.0204
39:	6.3921 eV	193.97 nm	f=0.0243
40:	6.4211 eV	193.09 nm	f=0.0180
41:	6.4612 eV	191.89 nm	f=0.0059
42:	6.4981 eV	190.80 nm	f=0.0196
43:	6.5332 eV	189.78 nm	f=0.0092

44:	6.5486	eV	189.33	nm	f=0.0097
45:	6.5948	eV	188.00	nm	f=0.0078
46:	6.6026	eV	187.78	nm	f=0.0082
47:	6.6317	eV	186.96	nm	f=0.0364
48:	6.6431	eV	186.64	nm	f=0.0023
49:	6.6987	eV	185.09	nm	f=0.0026
50:	6.7445	eV	183.83	nm	f=0.0319
51:	6.7634	eV	183.32	nm	f=0.0080
52:	6.7765	eV	182.96	nm	f=0.0054
53:	6.8153	eV	181.92	nm	f=0.0186
54:	6.8198	eV	181.80	nm	f=0.0123
55:	6.8333	eV	181.44	nm	f=0.0010
56:	6.8770	eV	180.29	nm	f=0.0001
57:	6.9056	eV	179.54	nm	f=0.0047
58:	6.9135	eV	179.34	nm	f=0.0052
59:	6.9269	eV	178.99	nm	f=0.0080
60:	6.9631	eV	178.06	nm	f=0.0006
61:	6.9820	eV	177.58	nm	f=0.0015
62:	6.9968	eV	177.20	nm	f=0.0067
63:	7.0348	eV	176.24	nm	f=0.0015
64:	7.0393	eV	176.13	nm	f=0.0060
65:	7.0856	eV	174.98	nm	f=0.0008
66:	7.1107	eV	174.36	nm	f=0.0111
67:	7.1609	eV	173.14	nm	f=0.0014
68:	7.1732	eV	172.84	nm	f=0.0080
69:	7.2118	eV	171.92	nm	f=0.0024
70:	7.2234	eV	171.64	nm	f=0.0017
71:	7.2512	eV	170.98	nm	f=0.0005
72:	7.2726	eV	170.48	nm	f=0.0027
73:	7.2942	eV	169.98	nm	f=0.0047
74:	7.3119	eV	169.57	nm	f=0.0208
75:	7.3483	eV	168.73	nm	f=0.0087
76:	7.3711	eV	168.20	nm	f=0.0218
77:	7.3997	eV	167.55	nm	f=0.0107
78:	7.4622	eV	166.15	nm	f=0.0100
79:	7.4866	eV	165.61	nm	f=0.0105
80:	7.5040	eV	165.22	nm	f=0.0053

$[Ti_3(\mu\text{-}(O)_3)]^{6+}$

State	Energy	Wavelength	Oscillator strength
1:	4.0866 eV	303.39 nm	f=0.0000
2:	4.0868 eV	303.38 nm	f=0.0000
3:	4.2190 eV	293.87 nm	f=0.0005
4:	4.3999 eV	281.79 nm	f=0.0000
5:	4.4480 eV	278.74 nm	f=0.0000
6:	4.4480 eV	278.74 nm	f=0.0000
7:	4.5642 eV	271.65 nm	f=0.0201
8:	4.5643 eV	271.64 nm	f=0.0201
9:	4.6074 eV	269.10 nm	f=0.0000
10:	4.8618 eV	255.02 nm	f=0.0000
11:	4.9056 eV	252.74 nm	f=0.0002
12:	4.9108 eV	252.47 nm	f=0.0016
13:	4.9109 eV	252.47 nm	f=0.0017
14:	4.9566 eV	250.14 nm	f=0.0000
15:	4.9570 eV	250.12 nm	f=0.0000
16:	4.9593 eV	250.01 nm	f=0.0000
17:	4.9795 eV	248.99 nm	f=0.0132
18:	4.9795 eV	248.99 nm	f=0.0131
19:	5.0444 eV	245.79 nm	f=0.0000
20:	5.1142 eV	242.43 nm	f=0.0000
21:	5.1588 eV	240.33 nm	f=0.0000
22:	5.1589 eV	240.33 nm	f=0.0000
23:	5.1664 eV	239.98 nm	f=0.0000
24:	5.1793 eV	239.38 nm	f=0.0935
25:	5.1795 eV	239.38 nm	f=0.0936
26:	5.2504 eV	236.14 nm	f=0.0000
27:	5.2506 eV	236.13 nm	f=0.0000
28:	5.2693 eV	235.29 nm	f=0.0000
29:	5.2763 eV	234.99 nm	f=0.0000
30:	5.3070 eV	233.62 nm	f=0.0070
31:	5.3072 eV	233.61 nm	f=0.0070
32:	5.3420 eV	232.09 nm	f=0.0000
33:	5.3518 eV	231.67 nm	f=0.0000
34:	5.3521 eV	231.66 nm	f=0.0000
35:	5.3706 eV	230.86 nm	f=0.0005
36:	5.4622 eV	226.99 nm	f=0.0708
37:	5.4622 eV	226.99 nm	f=0.0708
38:	5.5026 eV	225.32 nm	f=0.0000
39:	5.5028 eV	225.31 nm	f=0.0000
40:	5.5194 eV	224.63 nm	f=0.0015
41:	5.5226 eV	224.50 nm	f=0.0000
42:	5.5228 eV	224.50 nm	f=0.0000
43:	5.5820 eV	222.11 nm	f=0.1093

44:	5.6411	eV	219.79	nm	f=0.0000
45:	5.6506	eV	219.42	nm	f=0.0167
46:	5.6507	eV	219.41	nm	f=0.0167
47:	5.6692	eV	218.70	nm	f=0.0012
48:	5.6819	eV	218.21	nm	f=0.0000
49:	5.6832	eV	218.16	nm	f=0.0000
50:	5.6835	eV	218.15	nm	f=0.0000
51:	5.6970	eV	217.63	nm	f=0.0680
52:	5.6972	eV	217.62	nm	f=0.0680
53:	5.7839	eV	214.36	nm	f=0.0049
54:	5.7971	eV	213.87	nm	f=0.0000
55:	5.7973	eV	213.87	nm	f=0.0000
56:	5.8025	eV	213.67	nm	f=0.0075
57:	5.8028	eV	213.66	nm	f=0.0075
58:	5.8237	eV	212.90	nm	f=0.0000
59:	5.8238	eV	212.89	nm	f=0.0000
60:	5.8405	eV	212.28	nm	f=0.0876
61:	5.8548	eV	211.76	nm	f=0.0000
62:	5.8549	eV	211.76	nm	f=0.0000
63:	5.9234	eV	209.31	nm	f=0.0000
64:	5.9234	eV	209.31	nm	f=0.0000
65:	5.9347	eV	208.92	nm	f=0.0000
66:	5.9356	eV	208.88	nm	f=0.0000
67:	5.9607	eV	208.00	nm	f=0.0115
68:	5.9610	eV	207.99	nm	f=0.0115
69:	6.0200	eV	205.95	nm	f=0.0000
70:	6.0201	eV	205.95	nm	f=0.0000
71:	6.0367	eV	205.38	nm	f=0.0000
72:	6.0418	eV	205.21	nm	f=0.0006
73:	6.0689	eV	204.30	nm	f=0.0070
74:	6.0690	eV	204.29	nm	f=0.0070
75:	6.0784	eV	203.98	nm	f=0.0000
76:	6.1437	eV	201.81	nm	f=0.0000
77:	6.1496	eV	201.61	nm	f=0.0000
78:	6.1497	eV	201.61	nm	f=0.0000
79:	6.1580	eV	201.34	nm	f=0.0093
80:	6.1582	eV	201.33	nm	f=0.0093

$[Ti_2(\mu\text{-}O(OH))]^{5+}$ Canonical MOs depiction of two main excitations

Excited State 1 : 278 nm (4.45 eV)

HOMO - 4 -> LUMO	-0.13505
HOMO - 3 -> LUMO	-0.10148
HOMO - 2 -> LUMO	-0.25802
HOMO -> LUMO	0.57830
HOMO -> LUMO + 1	0.12692

Excited State 27 : 227 nm (5.462 eV)

HOMO - 10-> LUMO	0.11397
HOMO - 9 -> LUMO	0.15215
HOMO - 8 -> LUMO	-0.20703
HOMO - 6 -> LUMO + 1	-0.16257
HOMO - 6 -> LUMO +3	0.11338
HOMO - 5 -> LUMO + 1	-0.17725
HOMO - 5 -> LUMO + 2	0.23023
HOMO - 5 -> LUMO + 4	0.29707
HOMO - 4 -> LUMO + 2	-0.17204
HOMO - 4 -> LUMO + 3	-0.17813
HOMO - 3 -> LUMO + 2	0.12147
HOMO - 3 -> LUMO + 4	-0.12906
HOMO - 2 -> LUMO + 3	-0.11924

Representations of the orbitals involved in the electron transfer are shown in the following figures

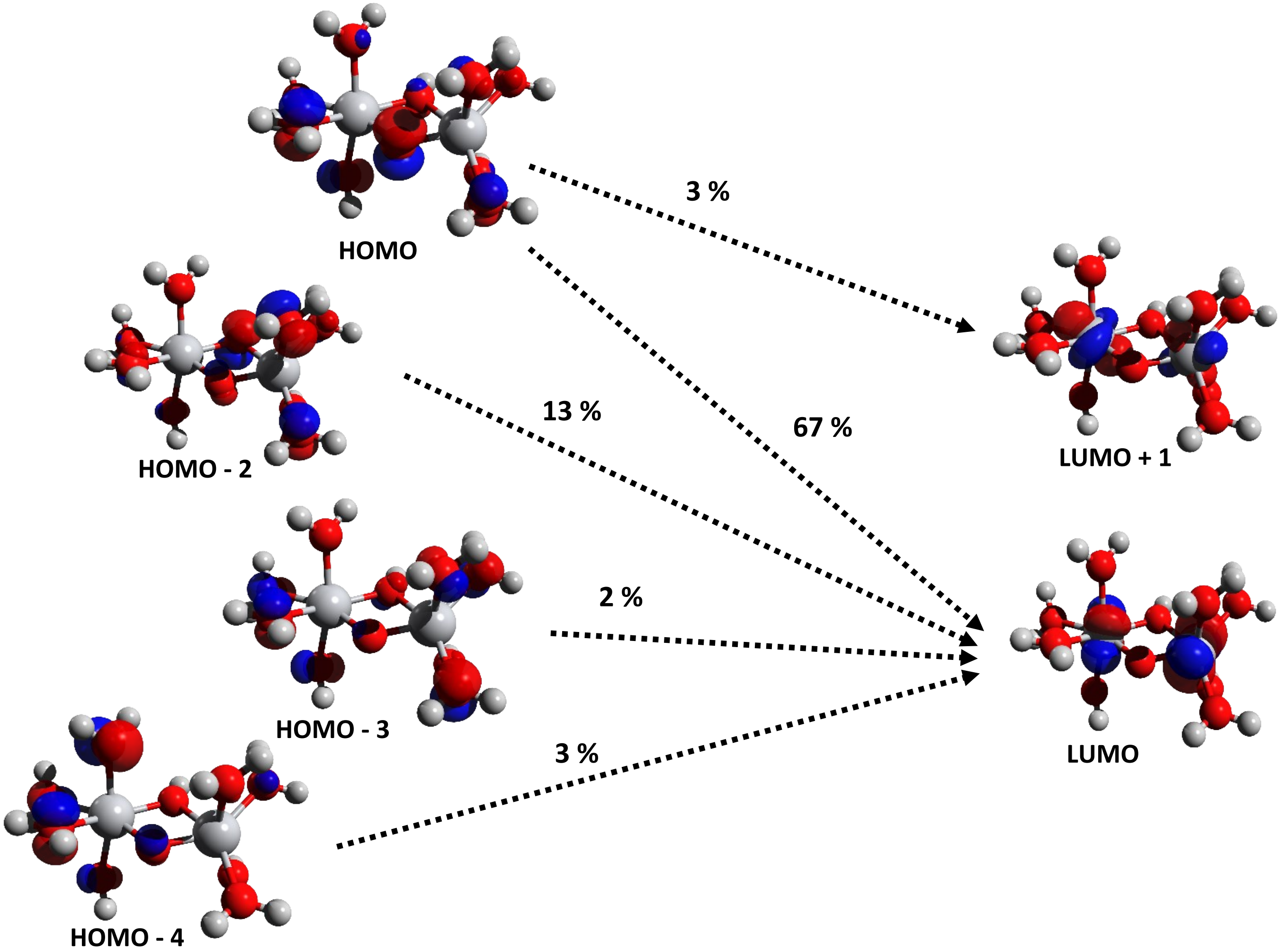

Excited State 1 : 278 nm (4.45 eV)

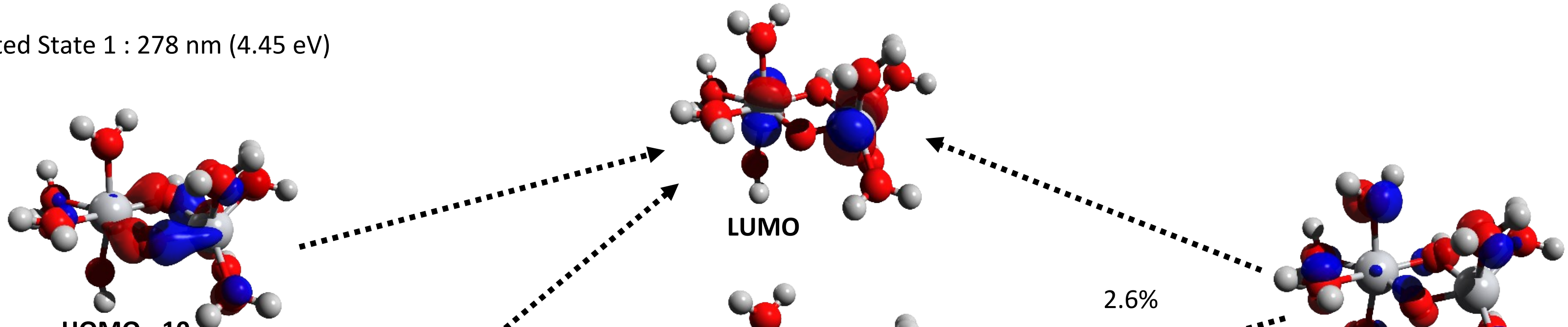

2.6%

8.6%

4.6%

5.3%

5.9%

6.3%

11%

6.4%

18%

3.0%

2.8%

3.3%

Excited State 27 : 227 nm (5.462 eV)
